# Supplementary figures and images for: A system for functional studies of the major virulence factor of malaria parasites
Source: eLife. 2025 Dec 29;13:RP103542. doi: 10.7554/eLife.103542 (PMC12747541; doi:10.7554/eLife.103542)

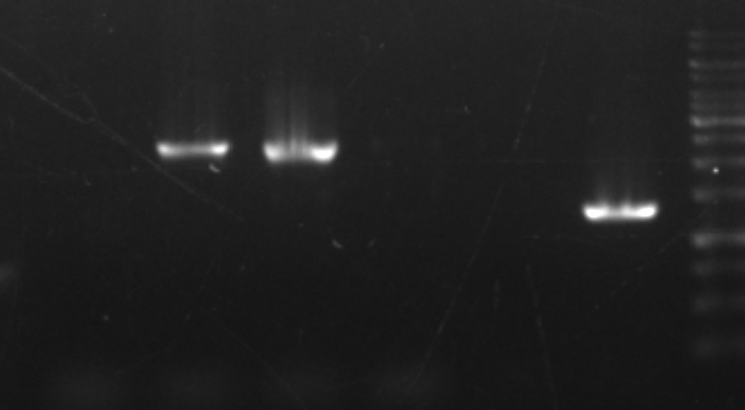

Supplement: Figure 1—source data 2. [file elife-103542-fig1-data2.zip › Figure 1-Source Data 2/raw agarose gels Fig1 BCGH/Agarose gel Figure 1B.tif]

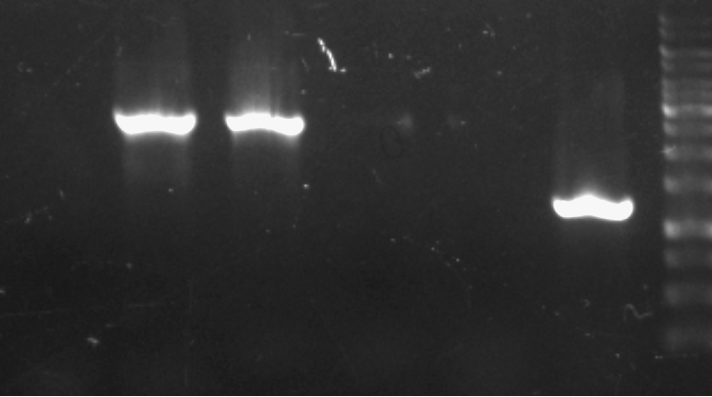

Supplement: Figure 1—source data 2. [file elife-103542-fig1-data2.zip › Figure 1-Source Data 2/raw agarose gels Fig1 BCGH/Agarose gel Figure 1C.tif]

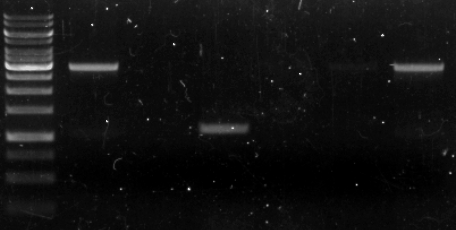

Supplement: Figure 1—source data 2. [file elife-103542-fig1-data2.zip › Figure 1-Source Data 2/raw agarose gels Fig1 BCGH/Agarose gel Figure 1G.tif]

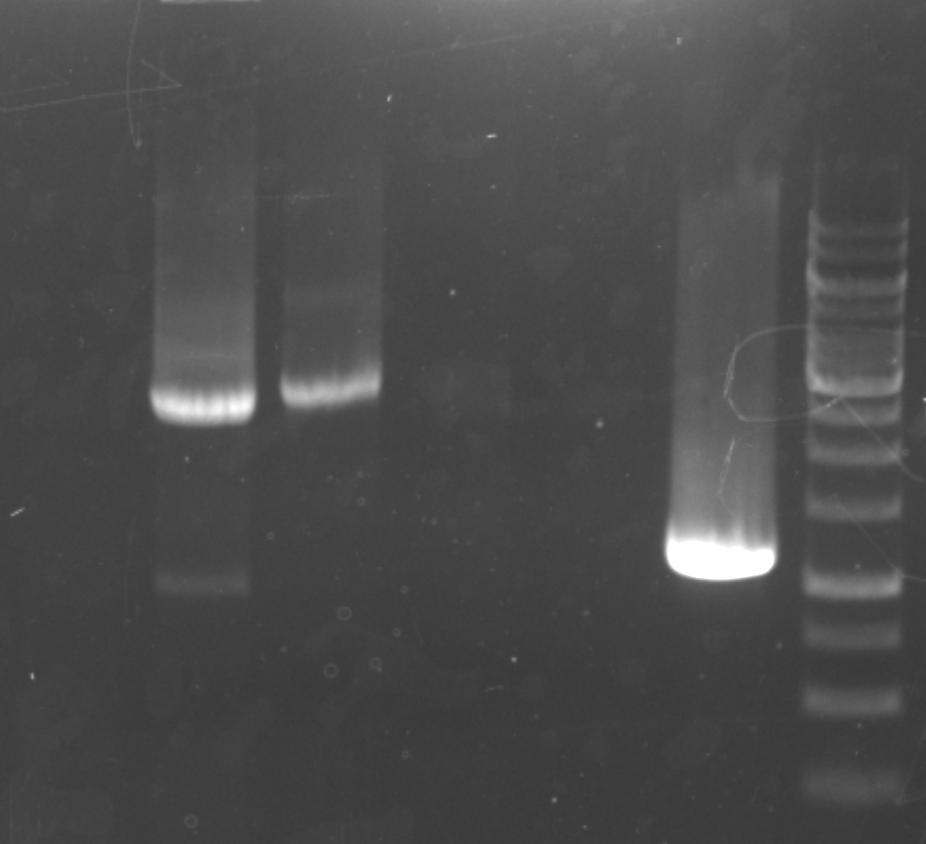

Supplement: Figure 1—source data 2. [file elife-103542-fig1-data2.zip › Figure 1-Source Data 2/raw agarose gels Fig1 BCGH/Agarose gel Figure 1H.tif]

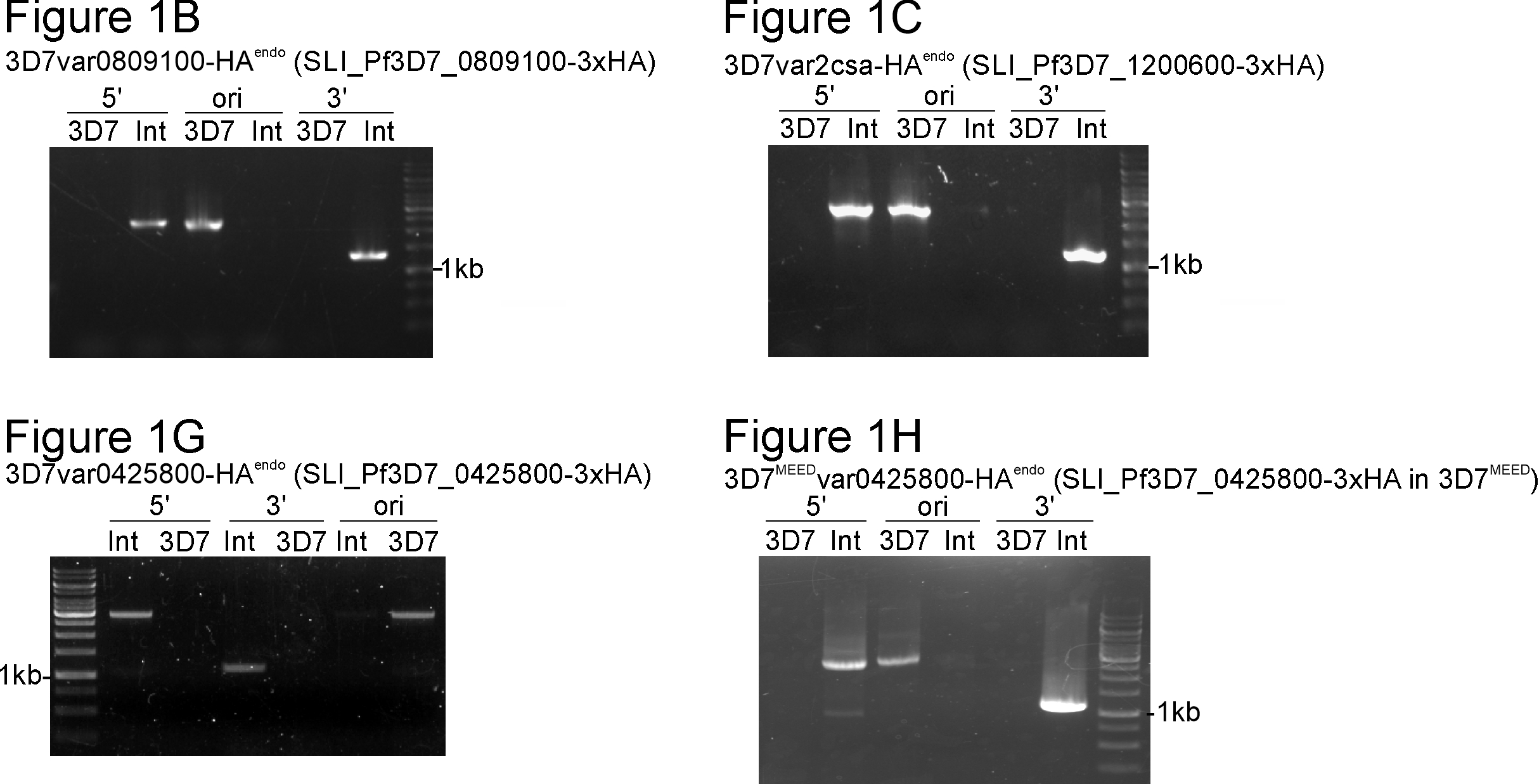

Supplement: Figure 1—source data 3. [file elife-103542-fig1-data3.zip › Figure 1-Source Data 3/Annotated agarose gels Fig1 BCGH/Figure 1BCGF_labelled.jpg]

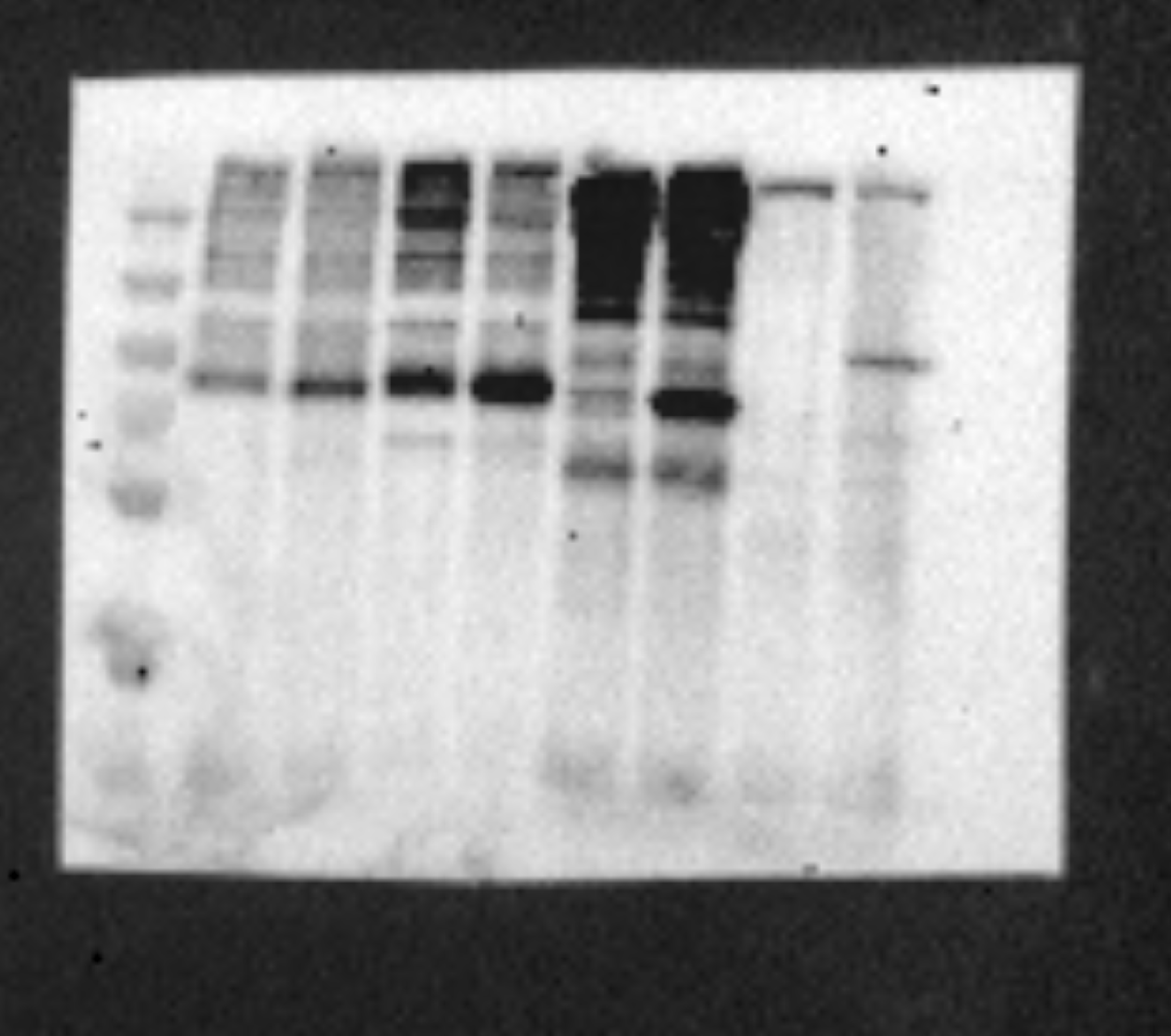

Supplement: Figure 1—source data 4. [file elife-103542-fig1-data4.zip › Figure 1-Source Data 4/raw blots Fig 1D/Figure 1D_blot_Var2CSA_HA_raw.tif]

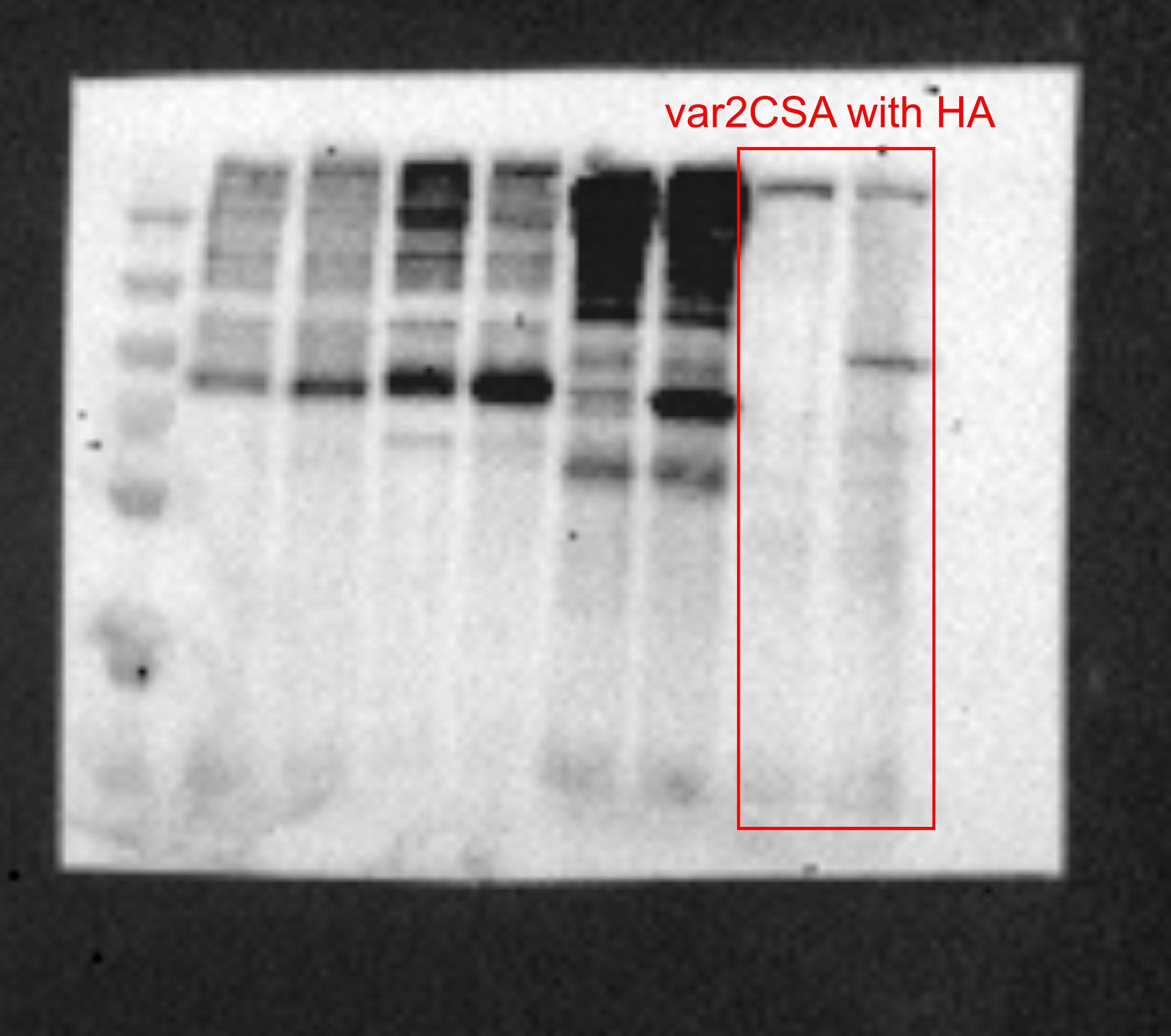

Supplement: Figure 1—source data 5. [file elife-103542-fig1-data5.zip › Figure 1-Source Data 5/annotated blots Fig 1D/Figure 1D_blot_Var2CSA_HA_labelled.tif]

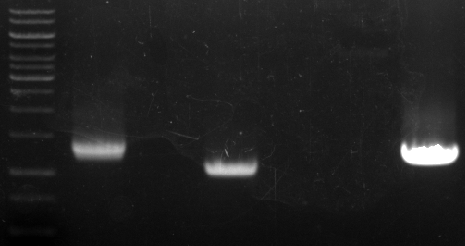

Supplement: Figure 1—figure supplement 1—source data 2. [file elife-103542-fig1-figsupp1-data2.zip › Figure 1-Figure supplement 1-Source Data 2/raw agarose gels Fig1-Fig-supplement1H/Agarose gel Figure 1-figure supplment1H rif0424700.tif]

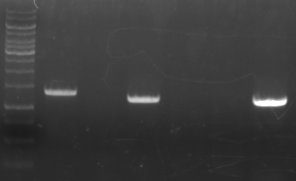

Supplement: Figure 1—figure supplement 1—source data 2. [file elife-103542-fig1-figsupp1-data2.zip › Figure 1-Figure supplement 1-Source Data 2/raw agarose gels Fig1-Fig-supplement1H/Agarose gel Figure 1-figure supplment1H rif254800.tif]

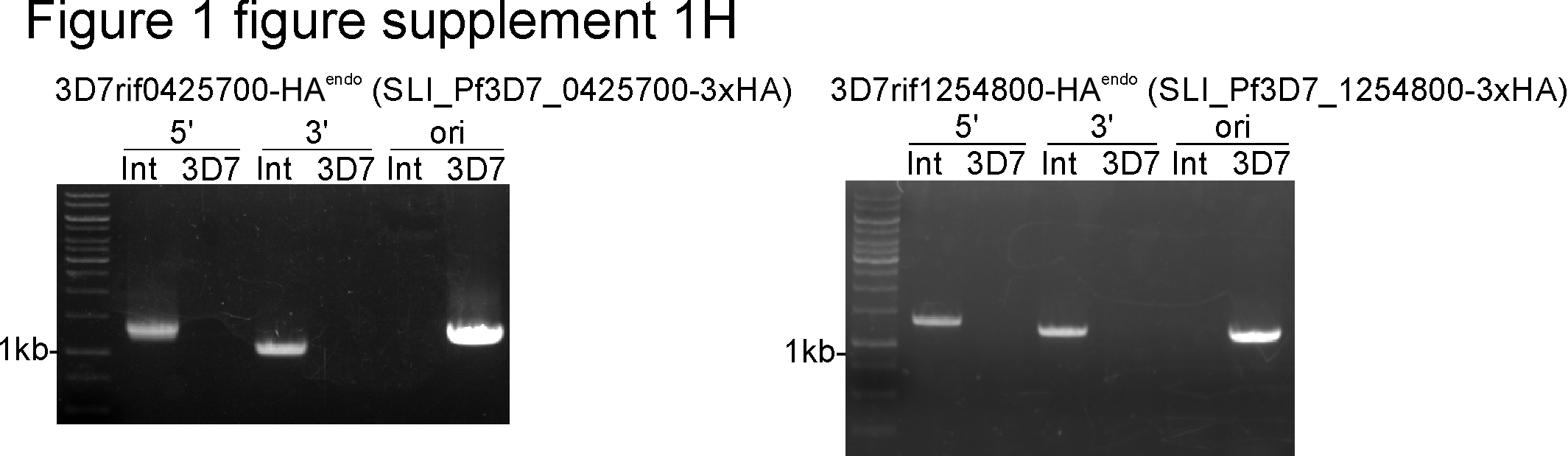

Supplement: Figure 1—figure supplement 1—source data 3. [file elife-103542-fig1-figsupp1-data3.zip › Figure 1-Figure supplement 1-Source Data 3/annotated agarose gels Fig1-Fig-supplement1H/Figure 1-figure supplement 1H_labelled.jpg]

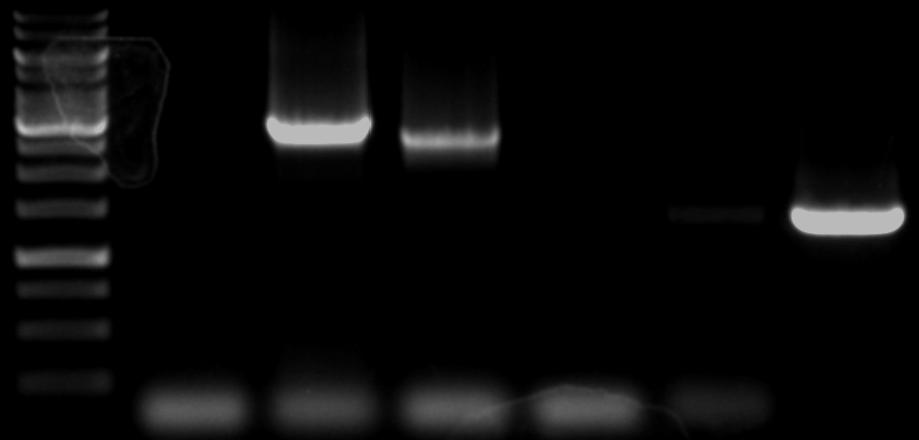

Supplement: Figure 2—figure supplement 1—source data 1. [file elife-103542-fig2-figsupp1-data1.zip › Figure 2-Figure supplement 1-Source Data 1/raw agarose gels Fig2/Agarose gel Figure 2-figure supplement1_var0809100-mDHFR.tif]

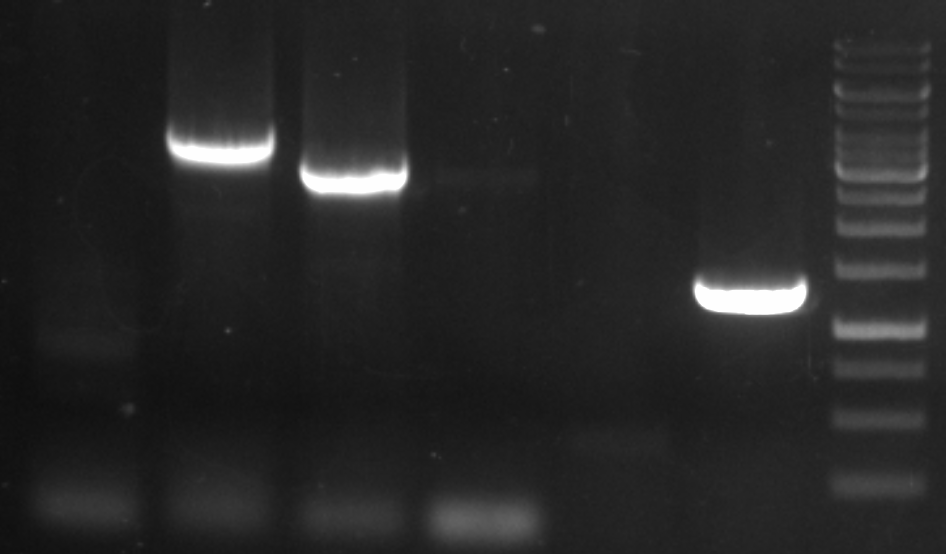

Supplement: Figure 2—figure supplement 1—source data 1. [file elife-103542-fig2-figsupp1-data1.zip › Figure 2-Figure supplement 1-Source Data 1/raw agarose gels Fig2/Agarose gel Figure 2-figure supplement1_var2csa-mDHFR.tif]

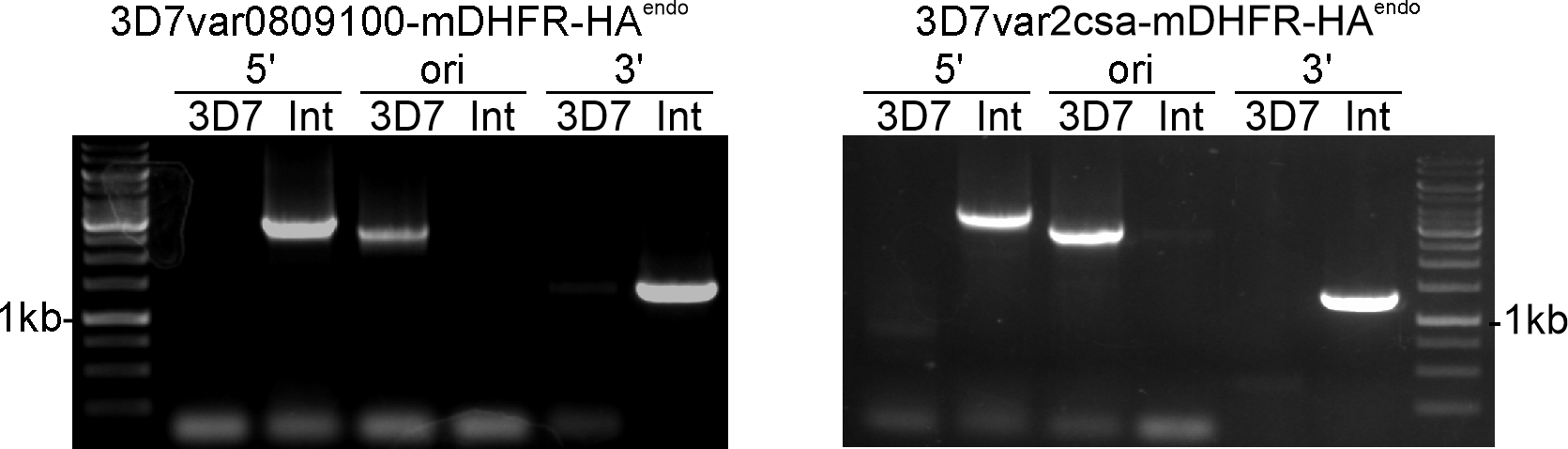

Supplement: Figure 2—figure supplement 1—source data 2. [file elife-103542-fig2-figsupp1-data2.zip › Figure 2-Figure supplement 1-Source Data 2/annotated agarose gels Fig2/Figure 2-figure supplement 1_labelled.jpg]

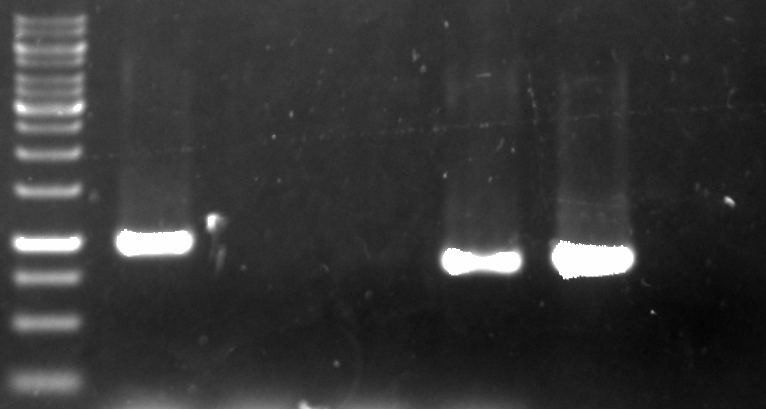

Supplement: Figure 3—source data 1. [file elife-103542-fig3-data1.zip › Figure 3-Source Data 1/raw agarose gels Fig 3AB/Agarose gel Figure 3A.tif]

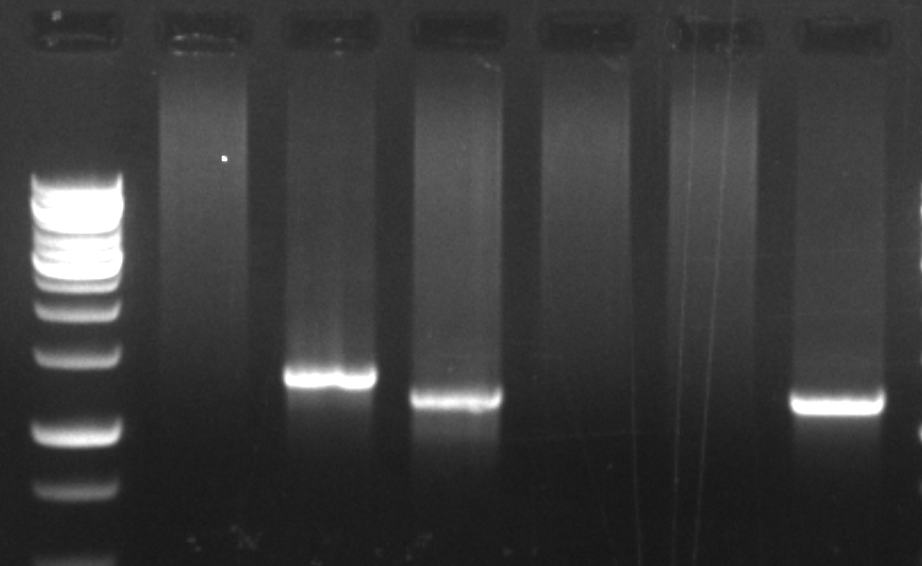

Supplement: Figure 3—source data 1. [file elife-103542-fig3-data1.zip › Figure 3-Source Data 1/raw agarose gels Fig 3AB/Agarose gel Figure 3B.tif]

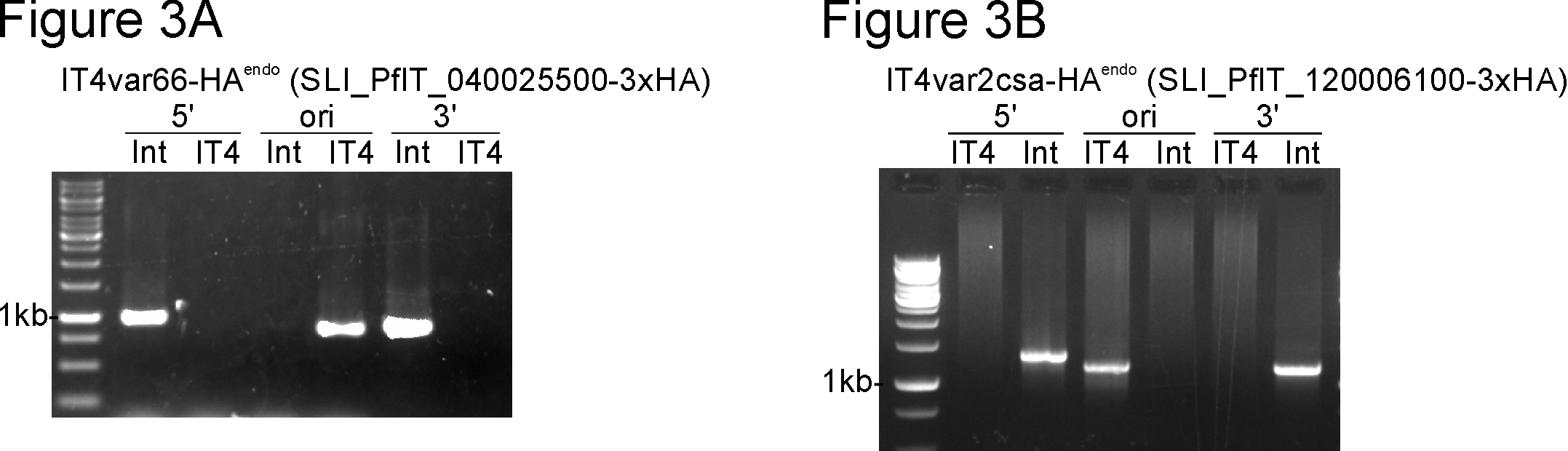

Supplement: Figure 3—source data 2. [file elife-103542-fig3-data2.zip › Figure 3-Source Data 2/annotated agarose gels Fig 3AB/Figure 3AB_labelled.jpg]

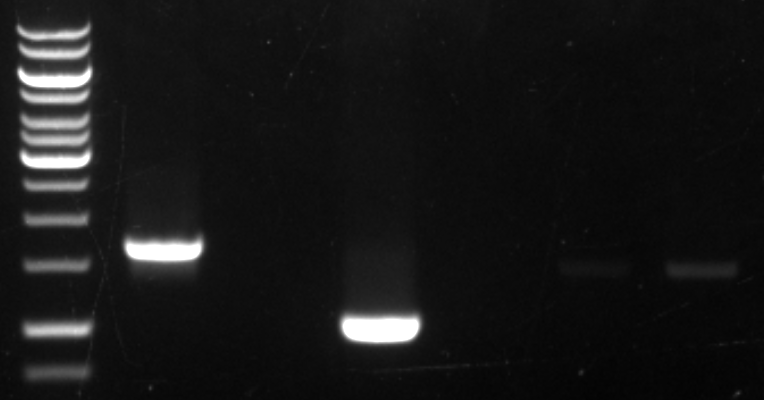

Supplement: Figure 4—source data 1. [file elife-103542-fig4-data1.zip › Figure 4-Source Data 1/raw agarose gels Fig 4A-C/Agarose gel Figure 4A.tif]

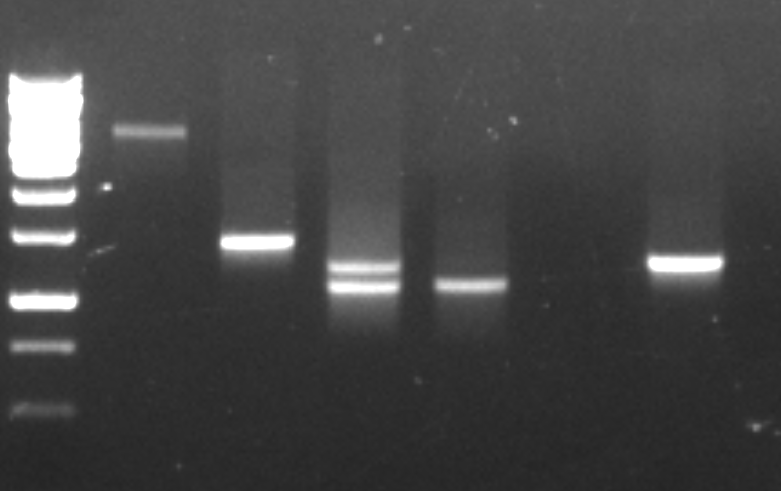

Supplement: Figure 4—source data 1. [file elife-103542-fig4-data1.zip › Figure 4-Source Data 1/raw agarose gels Fig 4A-C/Agarose gel Figure 4B.tif]

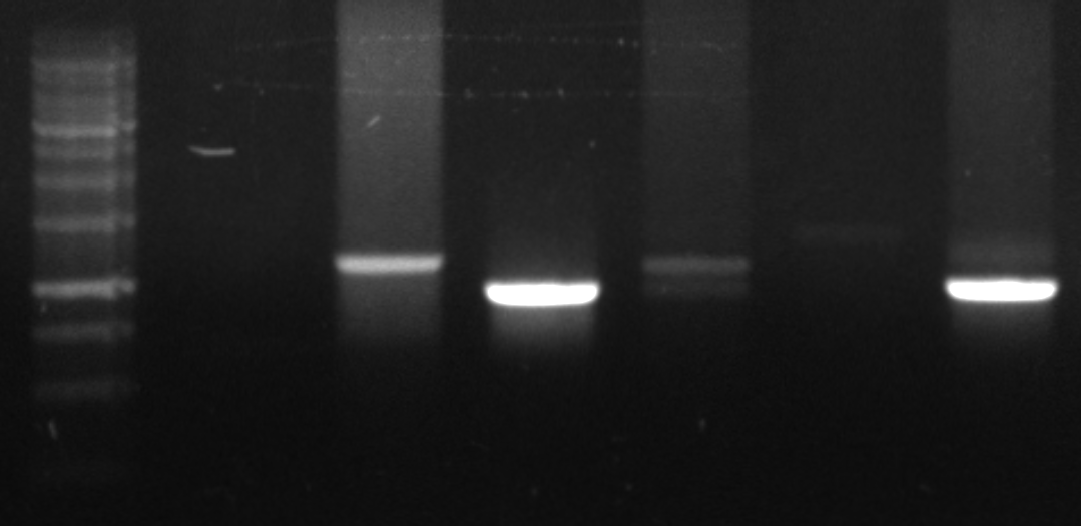

Supplement: Figure 4—source data 1. [file elife-103542-fig4-data1.zip › Figure 4-Source Data 1/raw agarose gels Fig 4A-C/Agarose gel Figure 4C.tif]

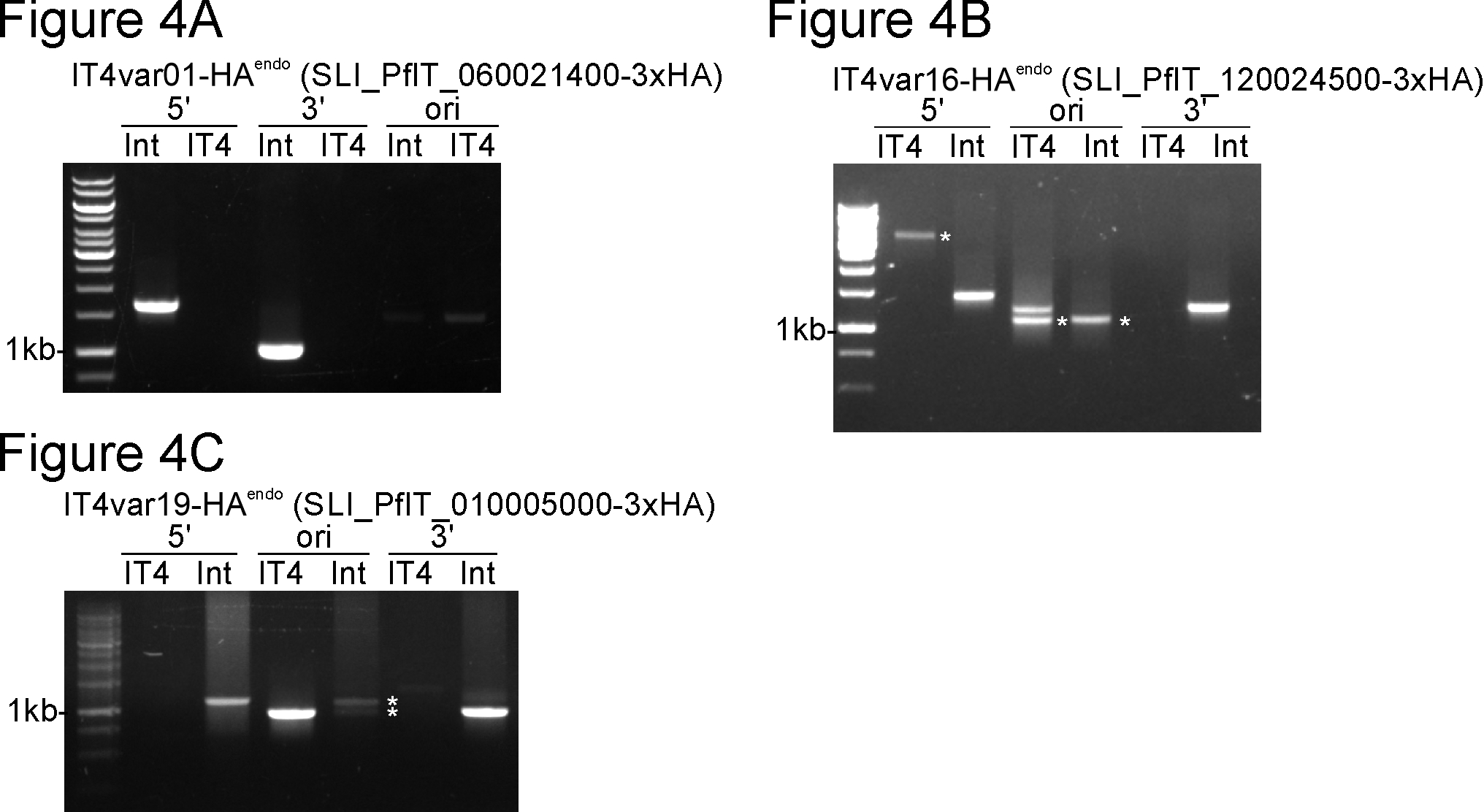

Supplement: Figure 4—source data 2. [file elife-103542-fig4-data2.zip › Figure 4-Source Data 2/annotated agarose gels Fig 4A-C/Figure 4ABC_labelled.jpg]

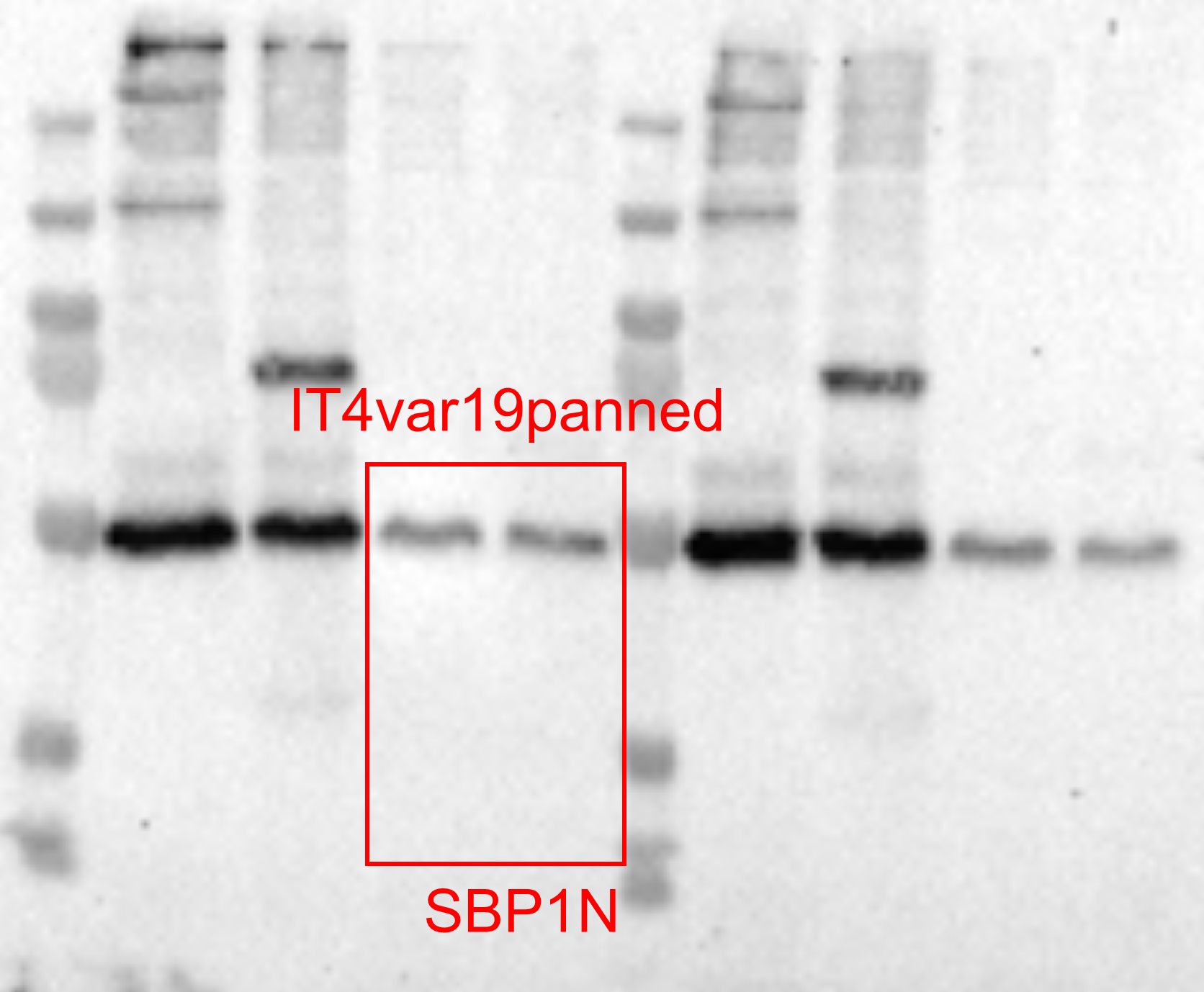

Supplement: Figure 4—source data 5. [file elife-103542-fig4-data5.zip › Figure 4-Source Data 5/marked/Figure 4H_SBP1N_marked.tif]

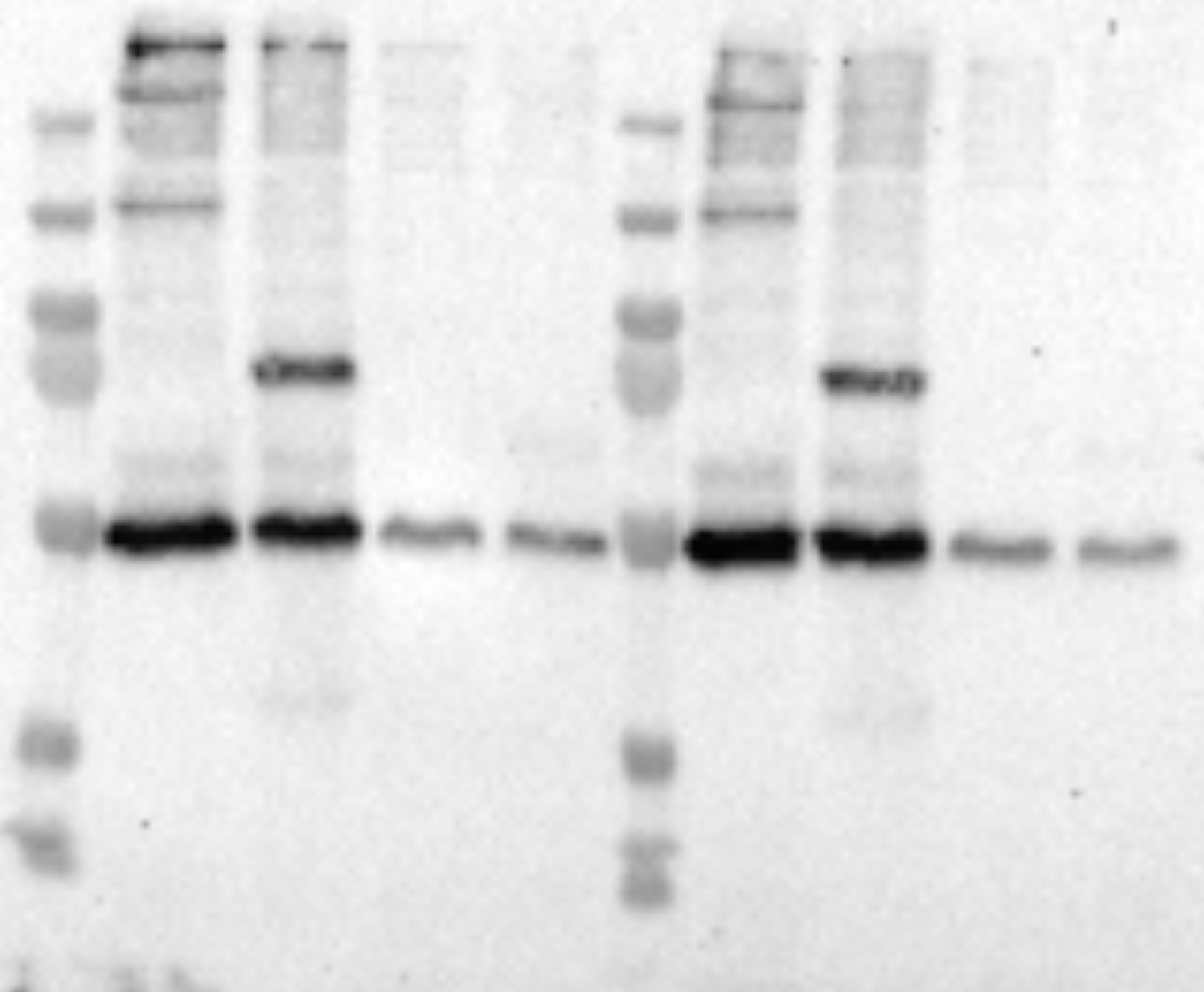

Supplement: Figure 4—source data 5. [file elife-103542-fig4-data5.zip › Figure 4-Source Data 5/raw/Figure 4H_SBP1N.tif]

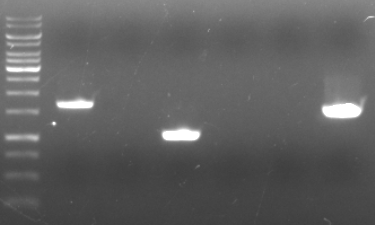

Supplement: Figure 5—source data 1. [file elife-103542-fig5-data1.zip › Figure 5-Source Data 1/raw agarose gels Fig 5B/Agarose gel Figure 5B_SLI1 integration.tif]

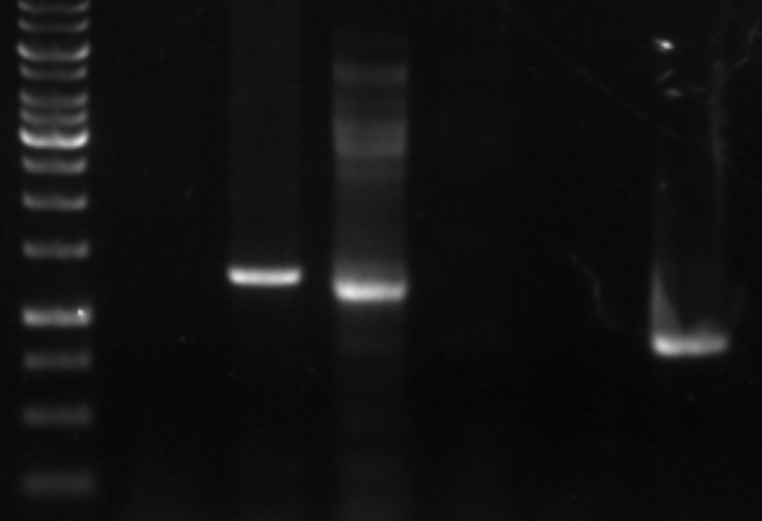

Supplement: Figure 5—source data 1. [file elife-103542-fig5-data1.zip › Figure 5-Source Data 1/raw agarose gels Fig 5B/Agarose gel Figure 5B_SLI2 integration.tif]

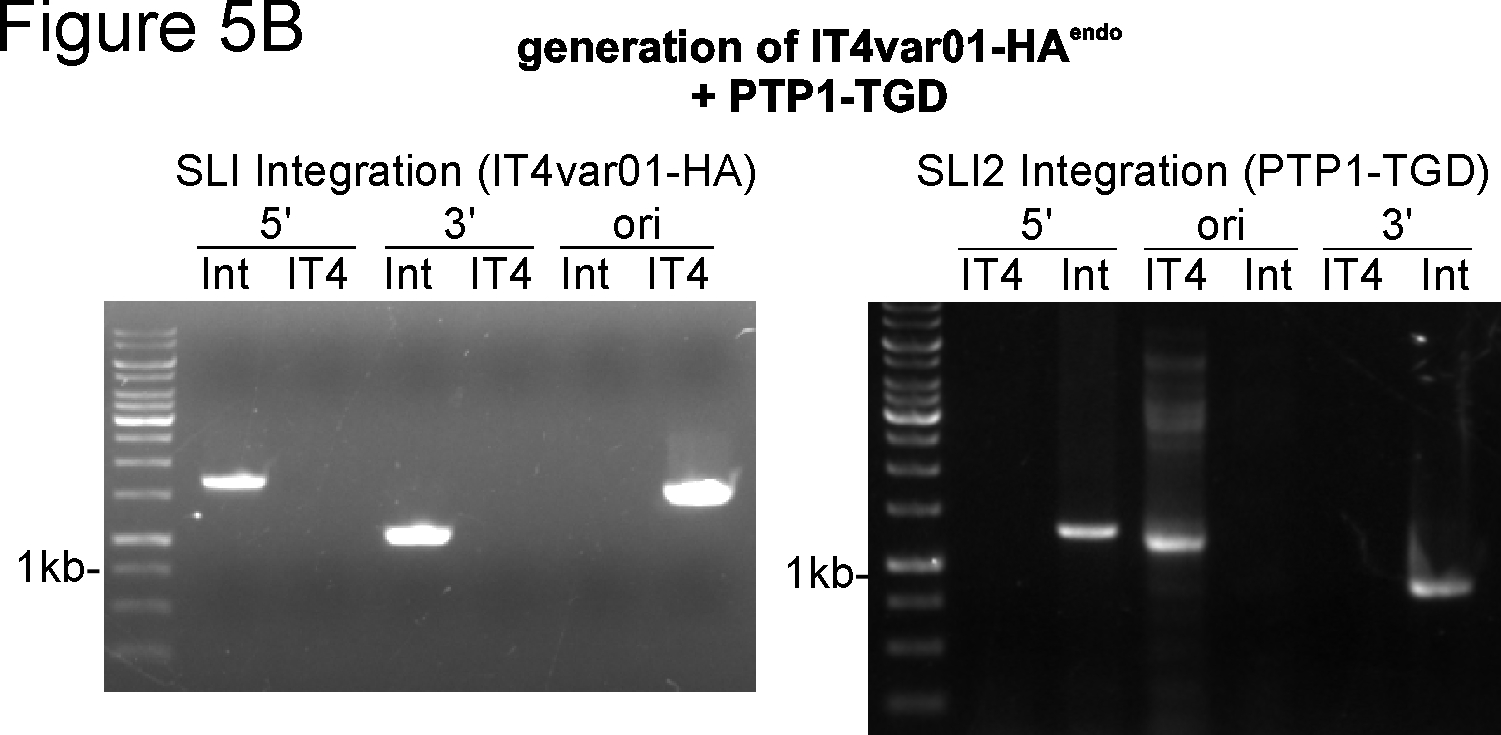

Supplement: Figure 5—source data 2. [file elife-103542-fig5-data2.zip › Figure 5-Source Data 2/annotated agarose gels Fig 5B/Figure 5B_labelled.jpg]

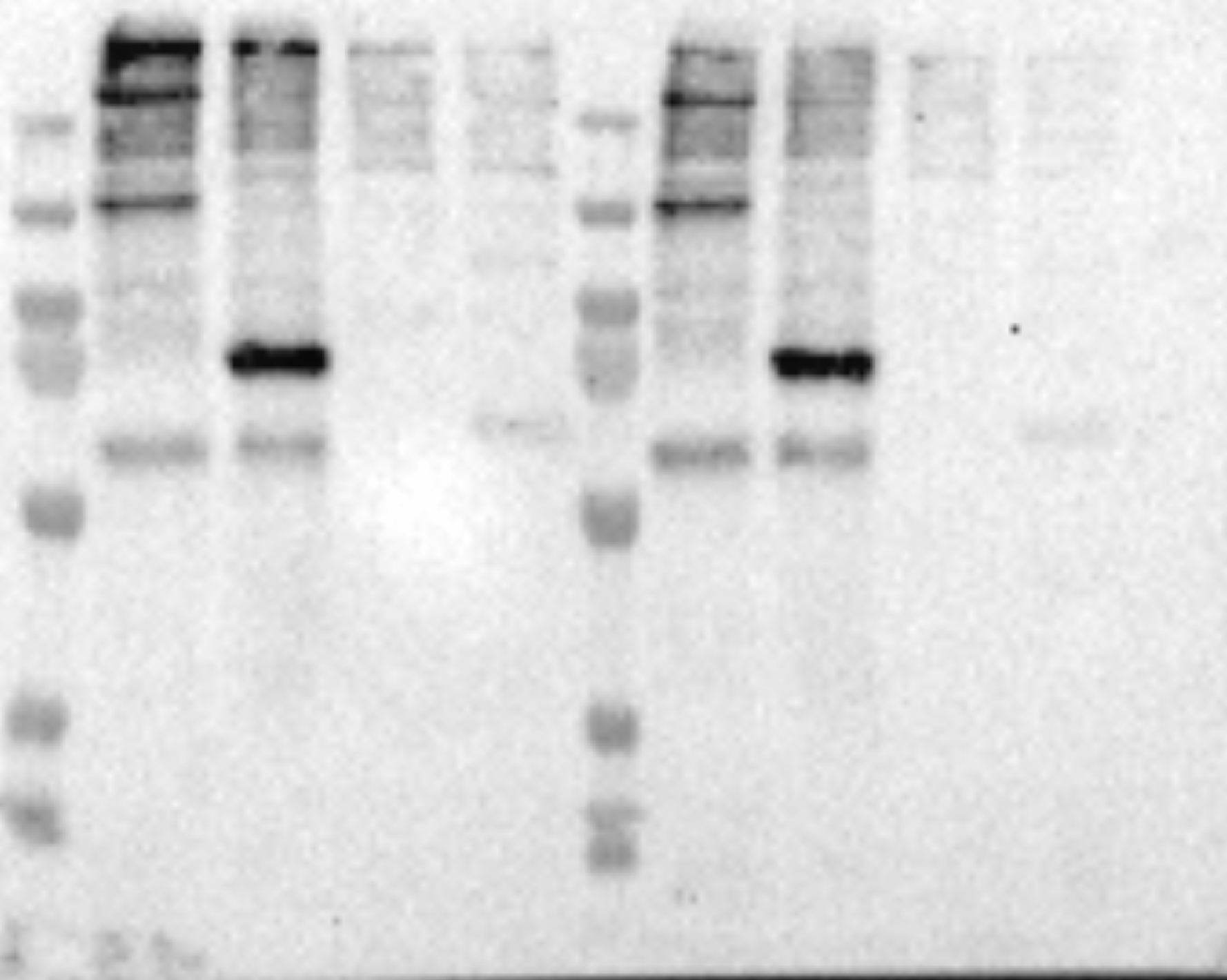

Supplement: Figure 6—source data 1. [file elife-103542-fig6-data1.zip › Figure 6-Source Data 1/raw blots Fig 6E/Figure 6E_IT4var01-BirA_Pos3_HA.tif]

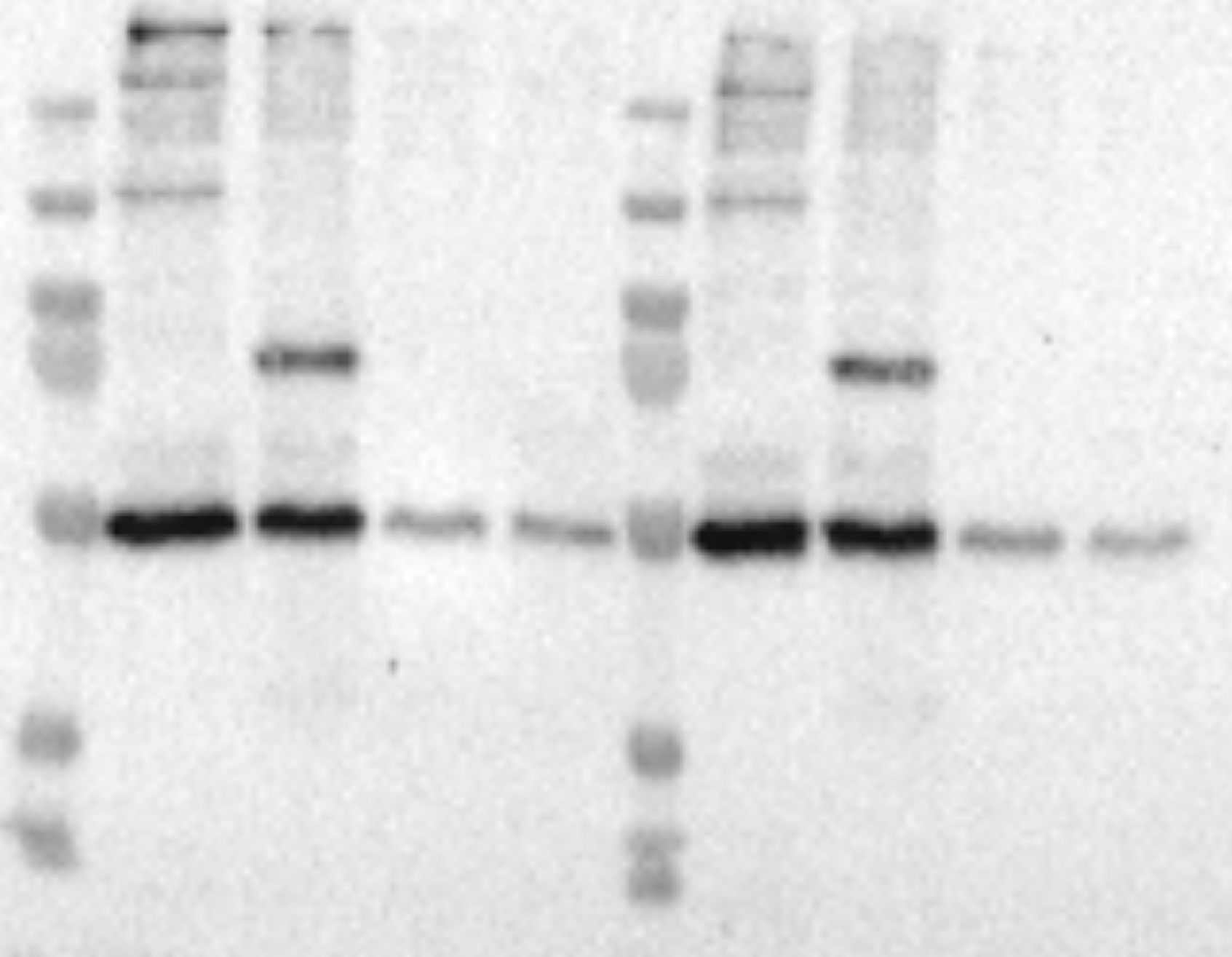

Supplement: Figure 6—source data 1. [file elife-103542-fig6-data1.zip › Figure 6-Source Data 1/raw blots Fig 6E/Figure 6E_IT4var01-BirA_Pos3_SBP1N.tif]

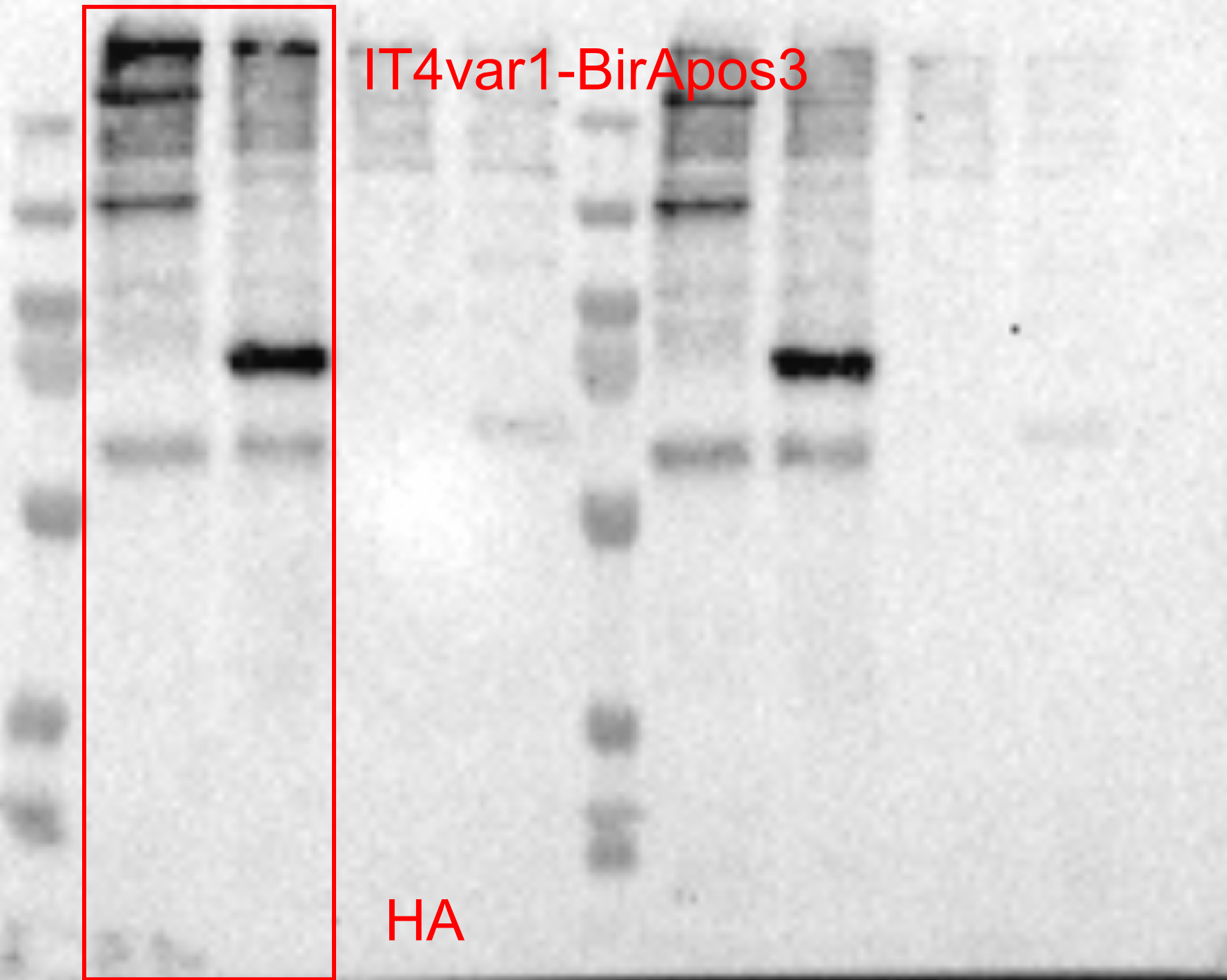

Supplement: Figure 6—source data 2. [file elife-103542-fig6-data2.zip › Figure 6-Source Data 2/marked blots Fig 6E/Figure 6E_IT4var01-BirA_Pos3_HA_marked.tif]

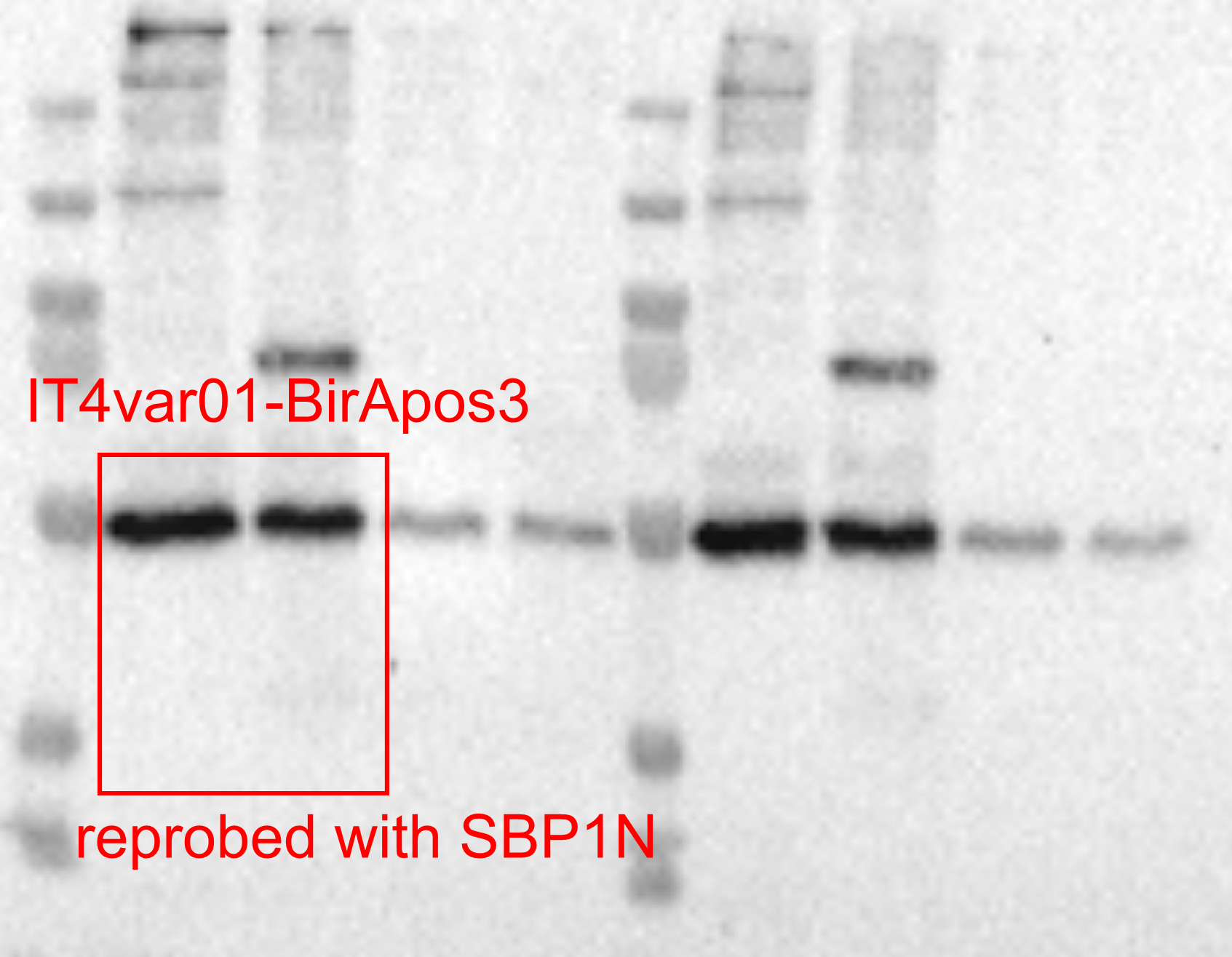

Supplement: Figure 6—source data 2. [file elife-103542-fig6-data2.zip › Figure 6-Source Data 2/marked blots Fig 6E/Figure 6E_IT4var01-BirA_Pos3_SBP1N_marked.tif]

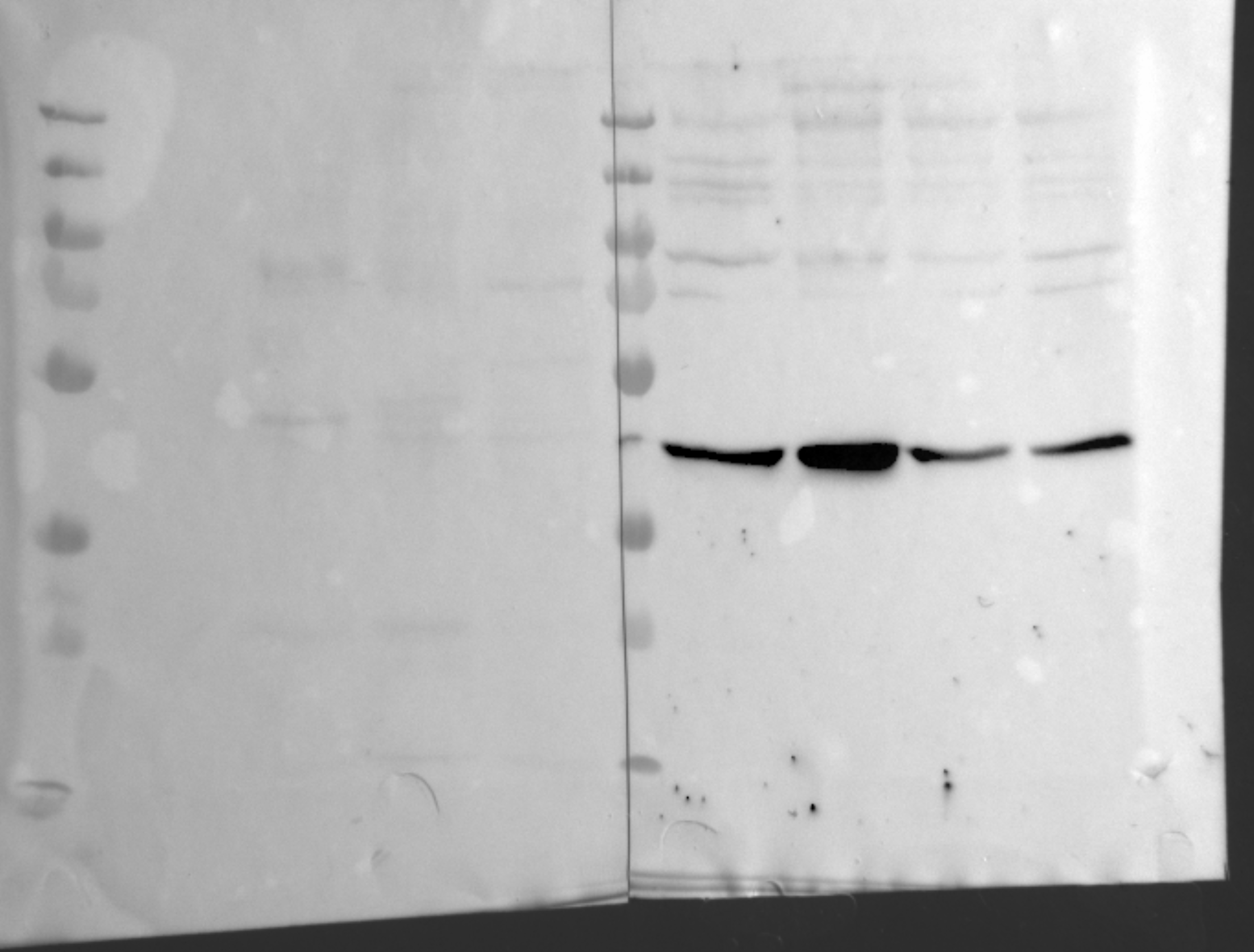

Supplement: Figure 6—source data 3. [file elife-103542-fig6-data3.zip › Figure 6-Source Data 3/raw blots Fig 6G/Figure 6G_Aldolase.tif]

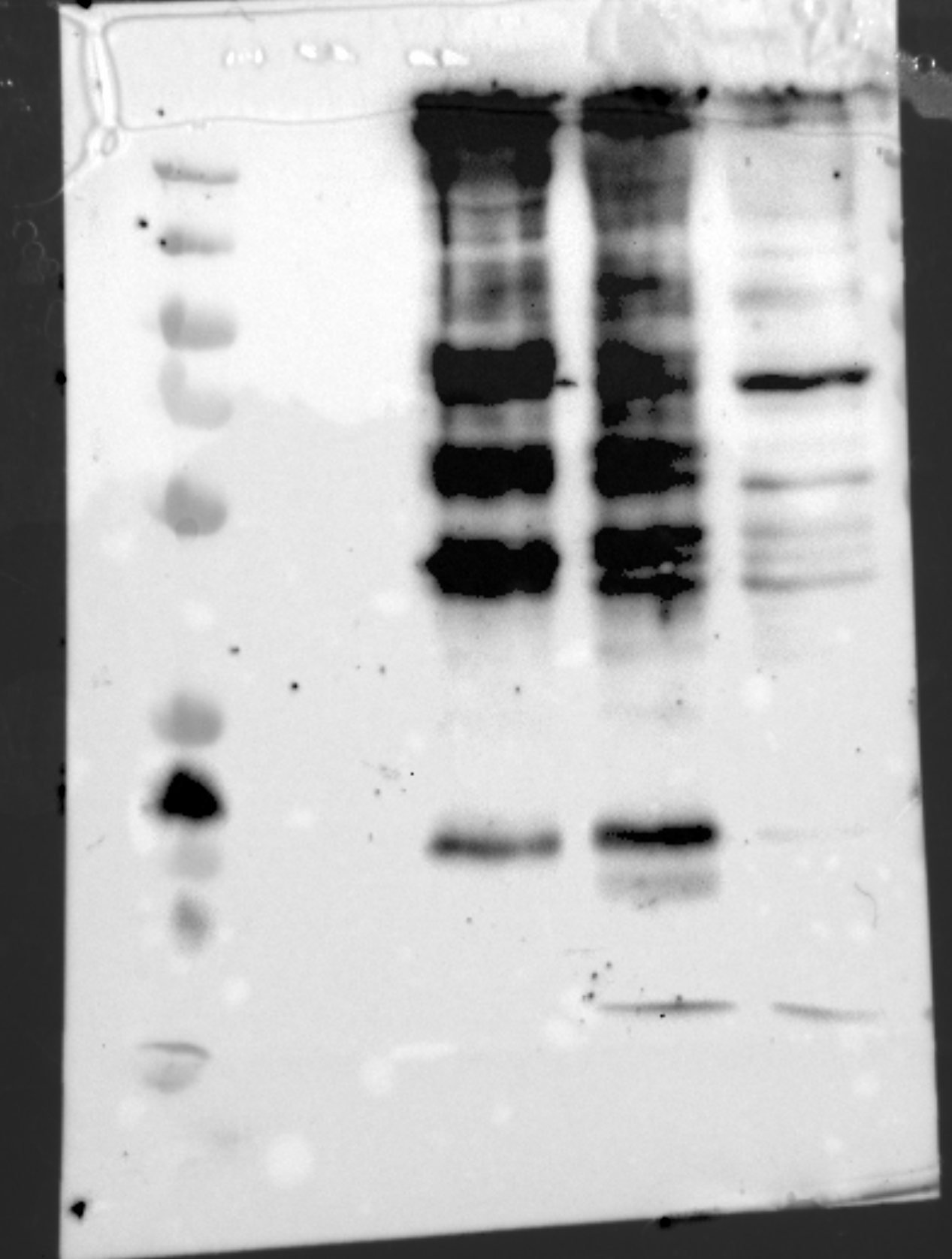

Supplement: Figure 6—source data 3. [file elife-103542-fig6-data3.zip › Figure 6-Source Data 3/raw blots Fig 6G/Figure 6G_Streptavidin-HRP.tif]

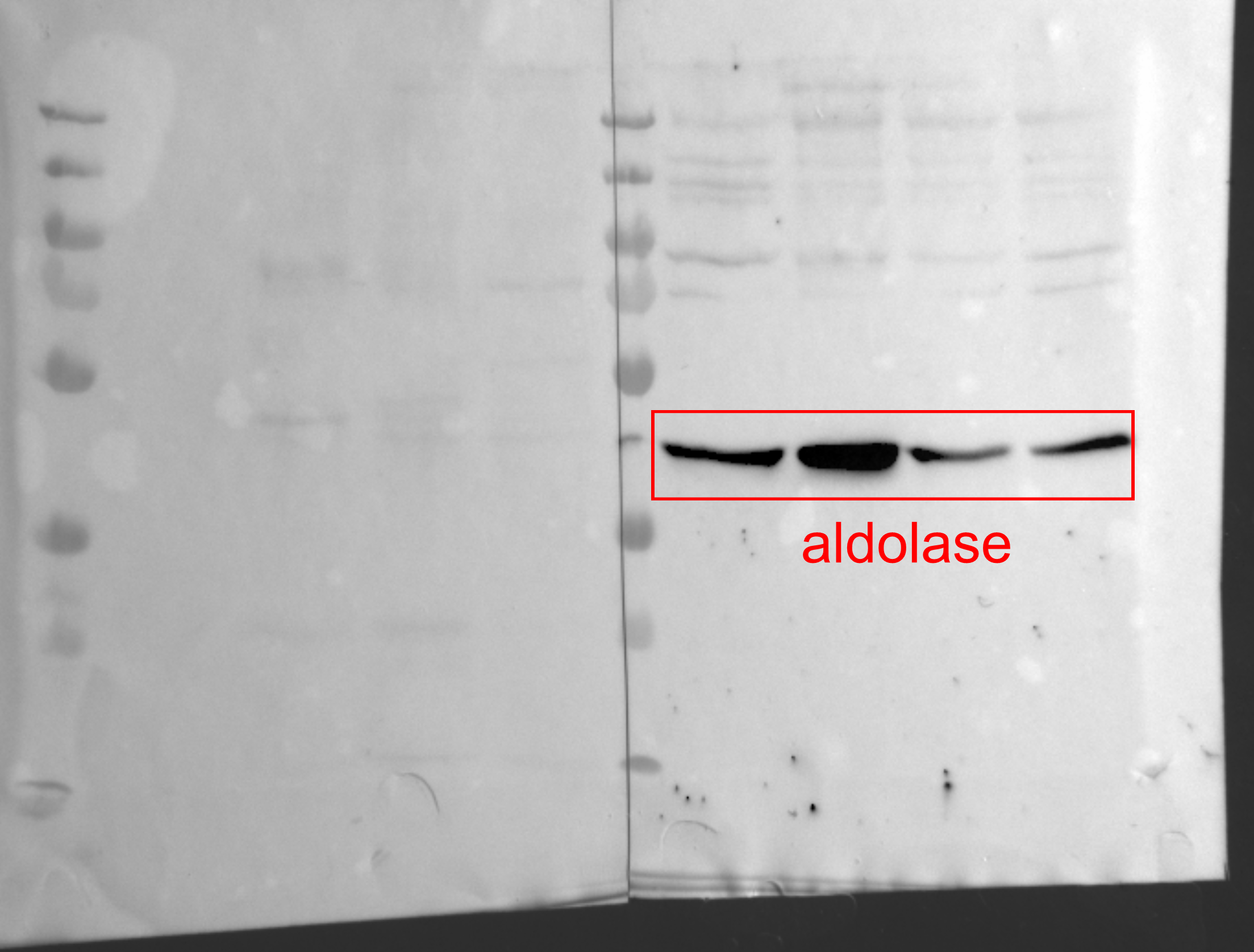

Supplement: Figure 6—source data 4. [file elife-103542-fig6-data4.zip › Figure 6-Source Data 4/marked blots Fig 6G/Figure 6G_Aldolase_marked.tif]

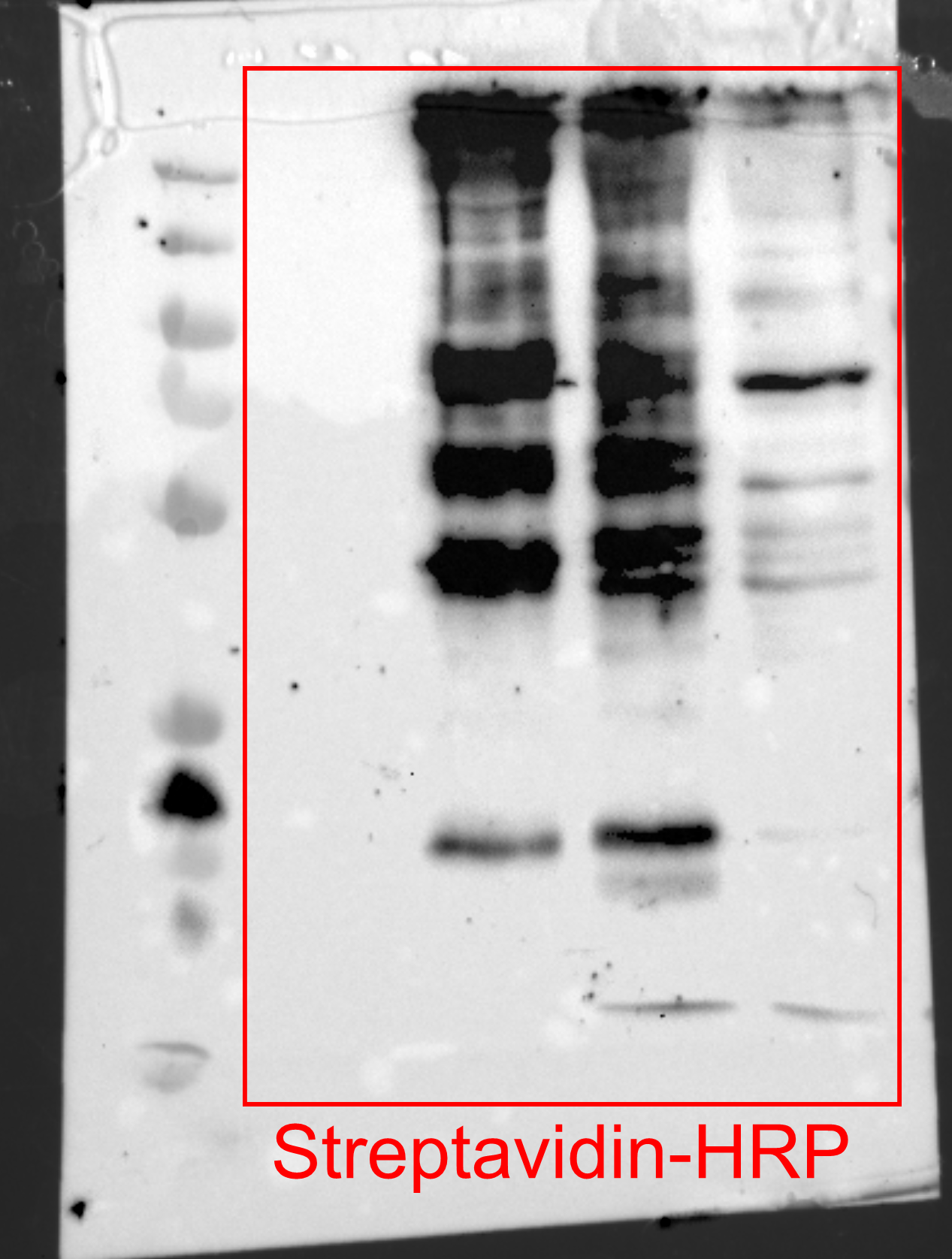

Supplement: Figure 6—source data 4. [file elife-103542-fig6-data4.zip › Figure 6-Source Data 4/marked blots Fig 6G/Figure 6G_Streptavidin-HRP_marked.tif]

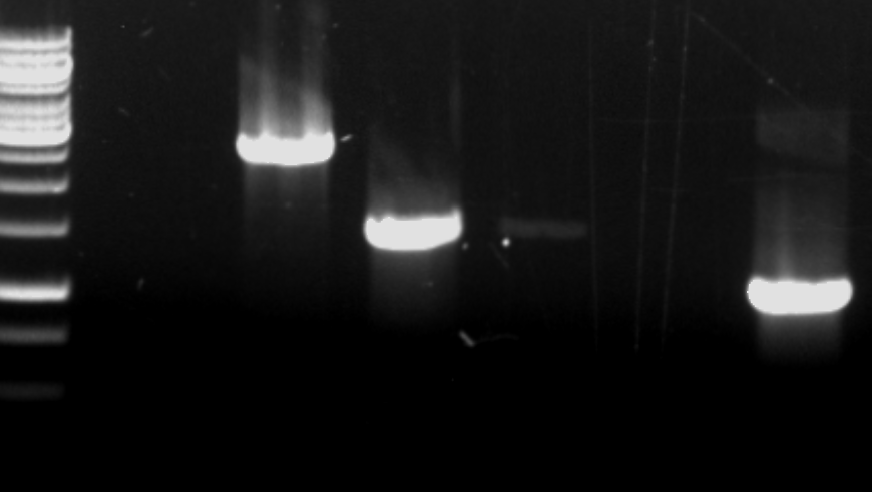

Supplement: Figure 6—figure supplement 1—source data 1. [file elife-103542-fig6-figsupp1-data1.zip › Figure 6-Figure Supplement 1-Source Data 1/raw agarose gels Fig6-supplement1 A-C/Agarose gel Figure 6-figure supplement1A.tif]

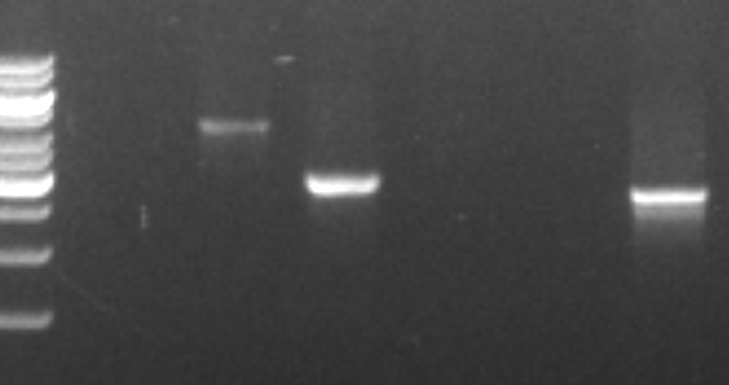

Supplement: Figure 6—figure supplement 1—source data 1. [file elife-103542-fig6-figsupp1-data1.zip › Figure 6-Figure Supplement 1-Source Data 1/raw agarose gels Fig6-supplement1 A-C/Agarose gel Figure 6-figure supplement1B.tif]

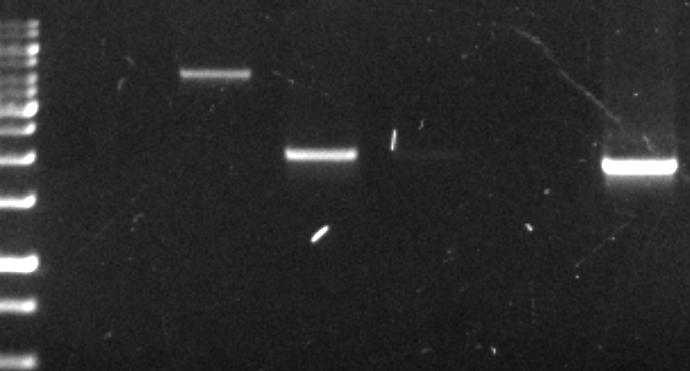

Supplement: Figure 6—figure supplement 1—source data 1. [file elife-103542-fig6-figsupp1-data1.zip › Figure 6-Figure Supplement 1-Source Data 1/raw agarose gels Fig6-supplement1 A-C/Agarose gel Figure 6-figure supplement1C.tif]

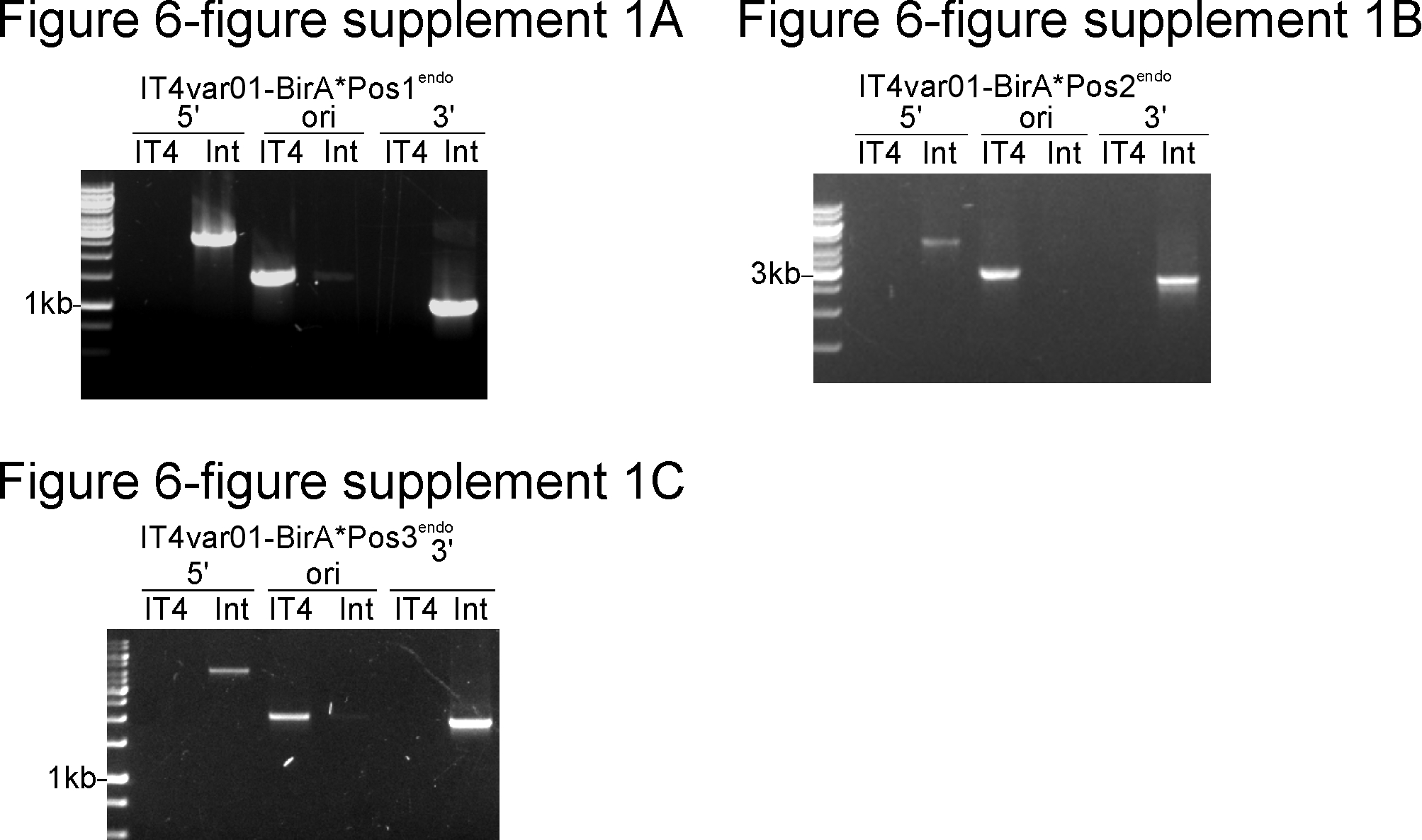

Supplement: Figure 6—figure supplement 1—source data 2. [file elife-103542-fig6-figsupp1-data2.zip › Figure 6-Figure Supplement 1-Source Data 2/annotated agarose gels Fig6-supplement1 A-C/Figure 6-figure supplement 1ABC_labelled.jpg]

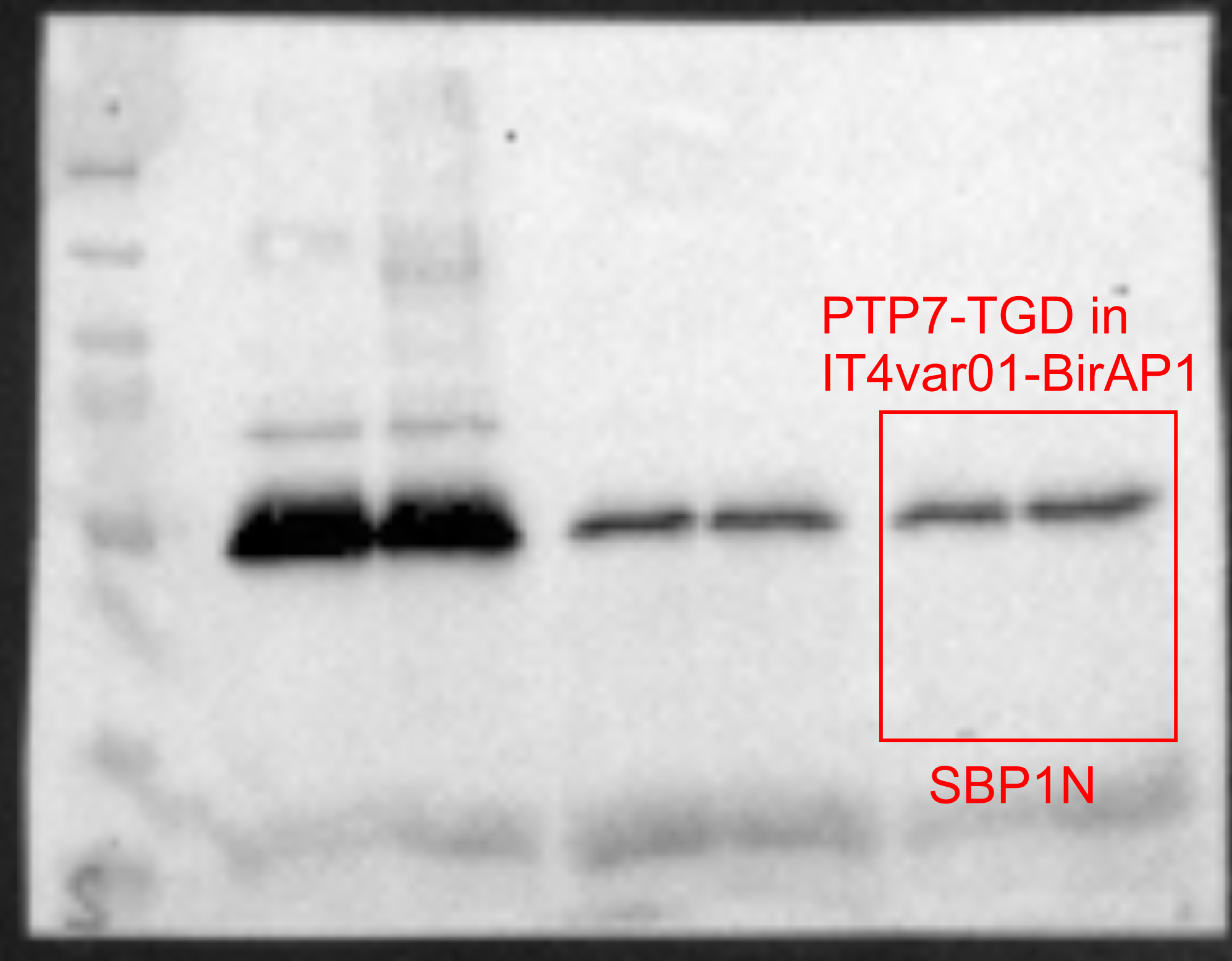

Supplement: Figure 7—source data 3. [file elife-103542-fig7-data3.zip › Figure 7-Source Data 2/marked blots Fig7 C/Figure 7C_PTP7-TGD in IT4var01P1_SBP1N_marked.tif]

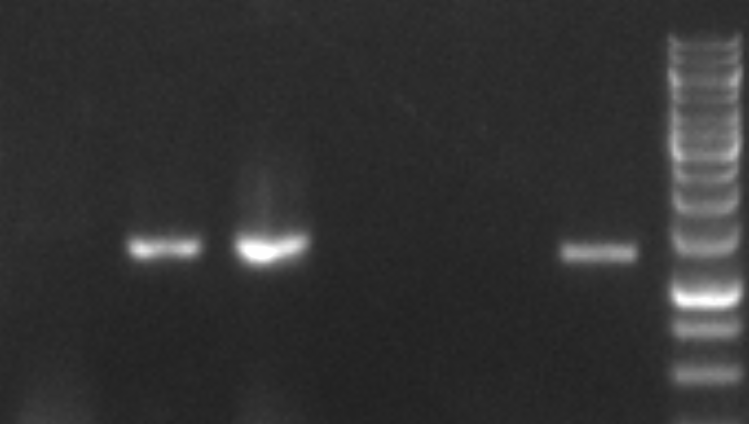

Supplement: Figure 7—figure supplement 1—source data 1. [file elife-103542-fig7-figsupp1-data1.zip › Figure 7-Figure Supplement 1-Source Data 1/raw agarose gels Fig7-supplement1 AB/Agarose gel Figure 7 - figure supplement 1A_EMPIC3-Ty.tif]

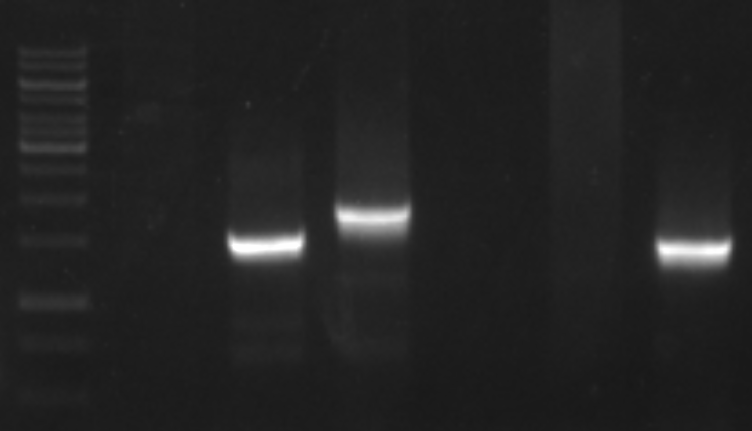

Supplement: Figure 7—figure supplement 1—source data 1. [file elife-103542-fig7-figsupp1-data1.zip › Figure 7-Figure Supplement 1-Source Data 1/raw agarose gels Fig7-supplement1 AB/Agarose gel Figure 7 - figure supplement 1A_PeMP2-Ty.tif]

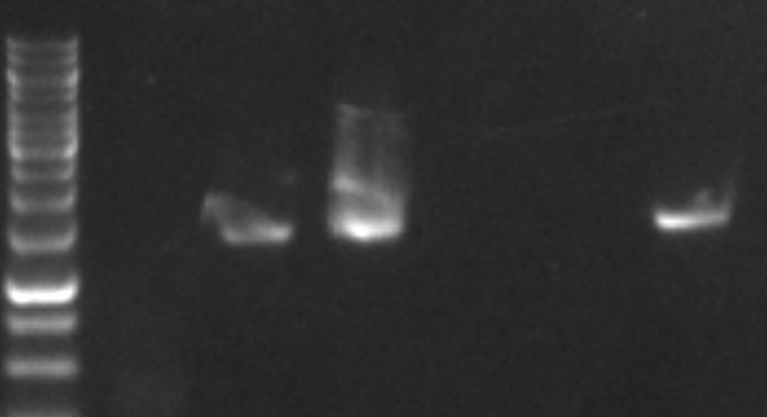

Supplement: Figure 7—figure supplement 1—source data 1. [file elife-103542-fig7-figsupp1-data1.zip › Figure 7-Figure Supplement 1-Source Data 1/raw agarose gels Fig7-supplement1 AB/Agarose gel Figure 7 - figure supplement 1A_PTEF-Ty.tif]

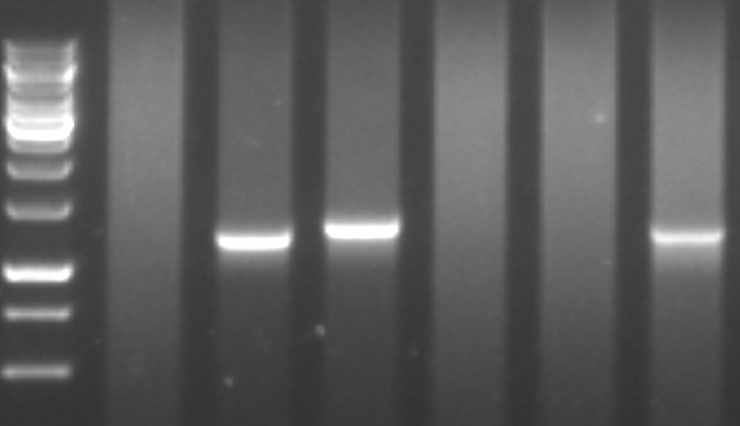

Supplement: Figure 7—figure supplement 1—source data 1. [file elife-103542-fig7-figsupp1-data1.zip › Figure 7-Figure Supplement 1-Source Data 1/raw agarose gels Fig7-supplement1 AB/Agarose gel Figure 7 - figure supplement 1A_TryThrA-Ty.tif]

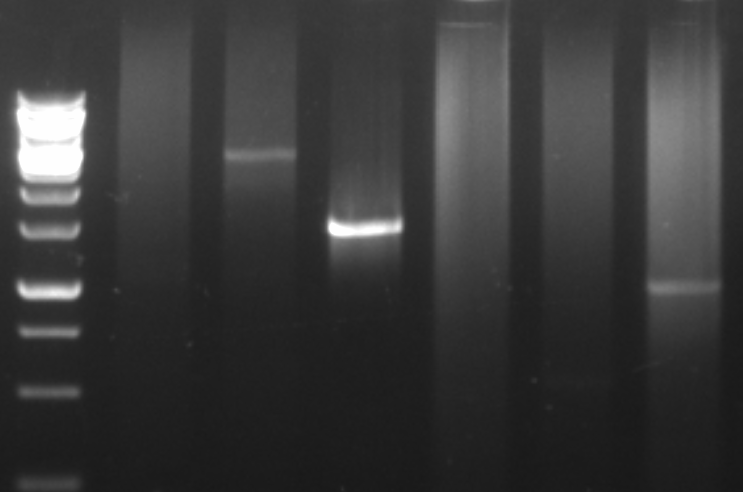

Supplement: Figure 7—figure supplement 1—source data 1. [file elife-103542-fig7-figsupp1-data1.zip › Figure 7-Figure Supplement 1-Source Data 1/raw agarose gels Fig7-supplement1 AB/Agarose gel Figure 7 - figure supplement 1B_EMPIC3-TGD in IT4var01-BirAP1_SLI1.tif]

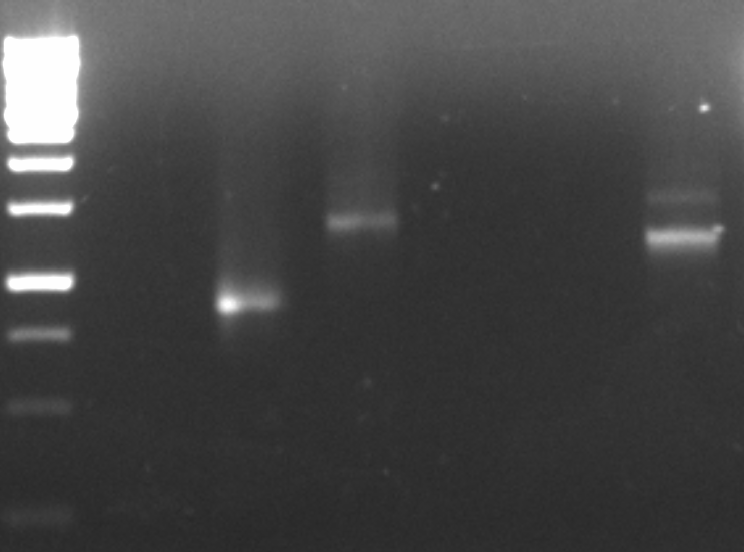

Supplement: Figure 7—figure supplement 1—source data 1. [file elife-103542-fig7-figsupp1-data1.zip › Figure 7-Figure Supplement 1-Source Data 1/raw agarose gels Fig7-supplement1 AB/Agarose gel Figure 7 - figure supplement 1B_EMPIC3-TGD in IT4var01-BirAP1_SLI2.tif]

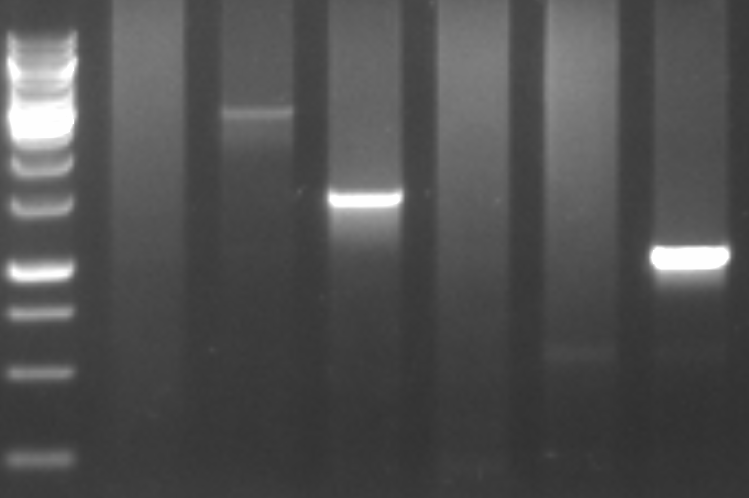

Supplement: Figure 7—figure supplement 1—source data 1. [file elife-103542-fig7-figsupp1-data1.zip › Figure 7-Figure Supplement 1-Source Data 1/raw agarose gels Fig7-supplement1 AB/Agarose gel Figure 7 - figure supplement 1B_PeMP2-TGD in IT4var01-BirAP1_SLI1.tif]

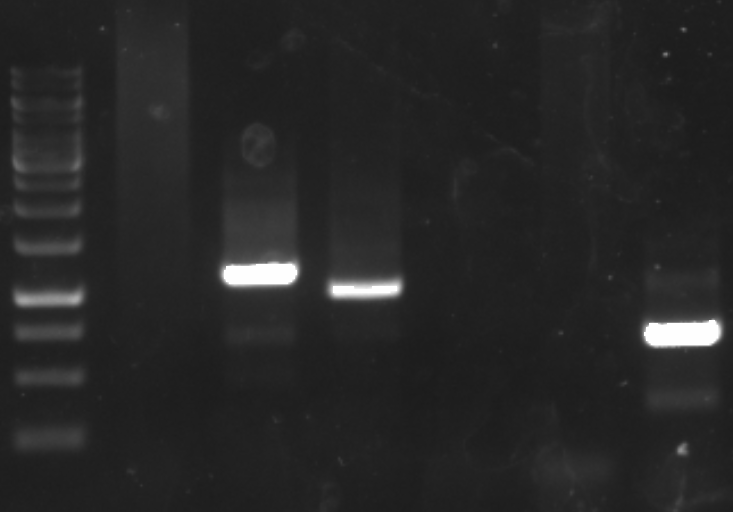

Supplement: Figure 7—figure supplement 1—source data 1. [file elife-103542-fig7-figsupp1-data1.zip › Figure 7-Figure Supplement 1-Source Data 1/raw agarose gels Fig7-supplement1 AB/Agarose gel Figure 7 - figure supplement 1B_PeMP2-TGD in IT4var01-BirAP1_SLI2.tif]

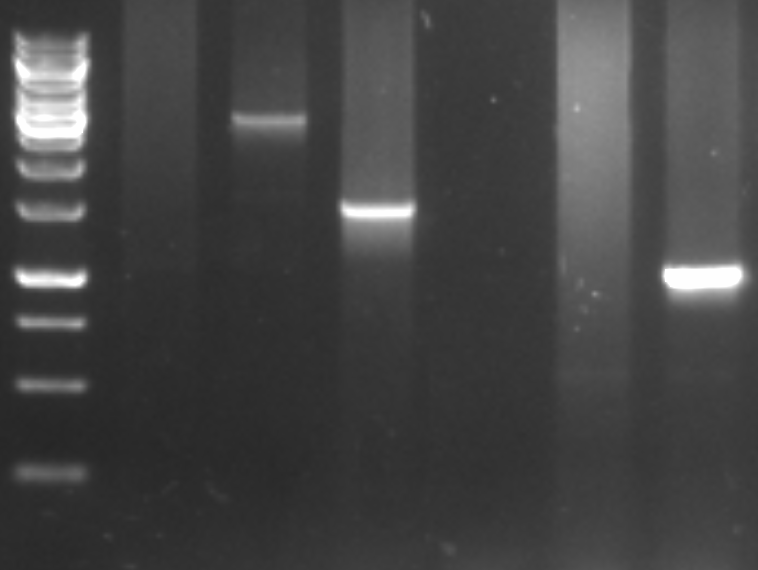

Supplement: Figure 7—figure supplement 1—source data 1. [file elife-103542-fig7-figsupp1-data1.zip › Figure 7-Figure Supplement 1-Source Data 1/raw agarose gels Fig7-supplement1 AB/Agarose gel Figure 7 - figure supplement 1B_PTEF-TGD in IT4var01-BirAP1_SLI1.tif]

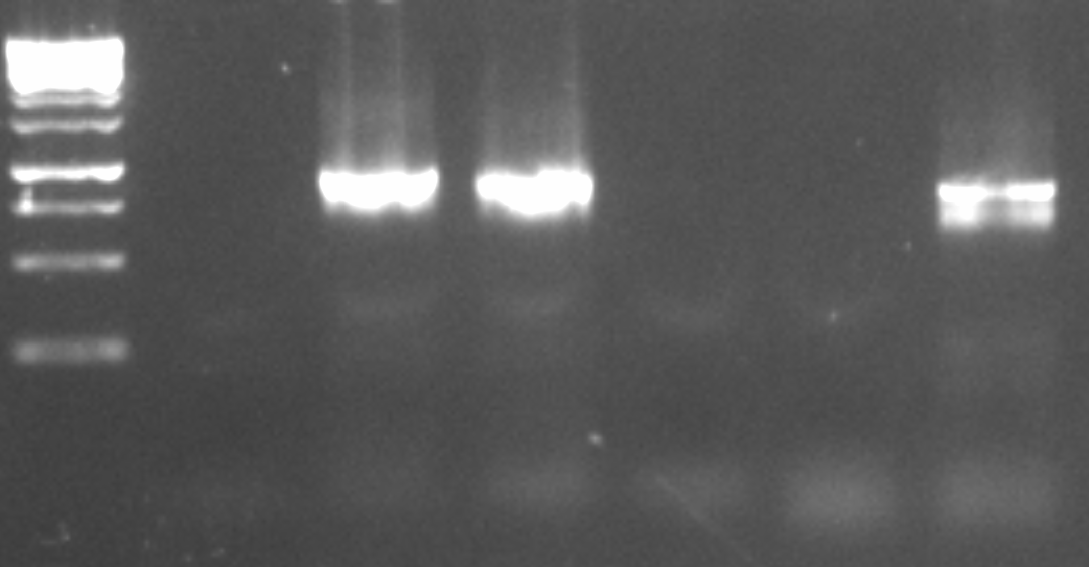

Supplement: Figure 7—figure supplement 1—source data 1. [file elife-103542-fig7-figsupp1-data1.zip › Figure 7-Figure Supplement 1-Source Data 1/raw agarose gels Fig7-supplement1 AB/Agarose gel Figure 7 - figure supplement 1B_PTEF-TGD in IT4var01-BirAP1_SLI2.tif]

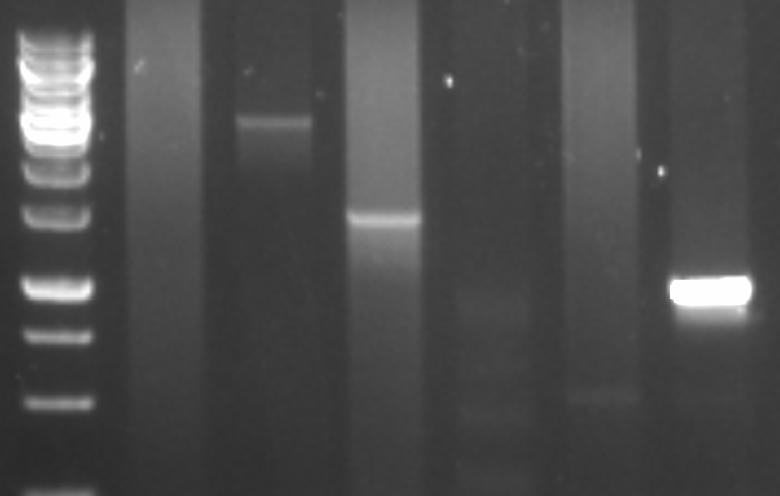

Supplement: Figure 7—figure supplement 1—source data 1. [file elife-103542-fig7-figsupp1-data1.zip › Figure 7-Figure Supplement 1-Source Data 1/raw agarose gels Fig7-supplement1 AB/Agarose gel Figure 7 - figure supplement 1B_PTP7-TGD in IT4var01-BirAP1_SLI1.tif]

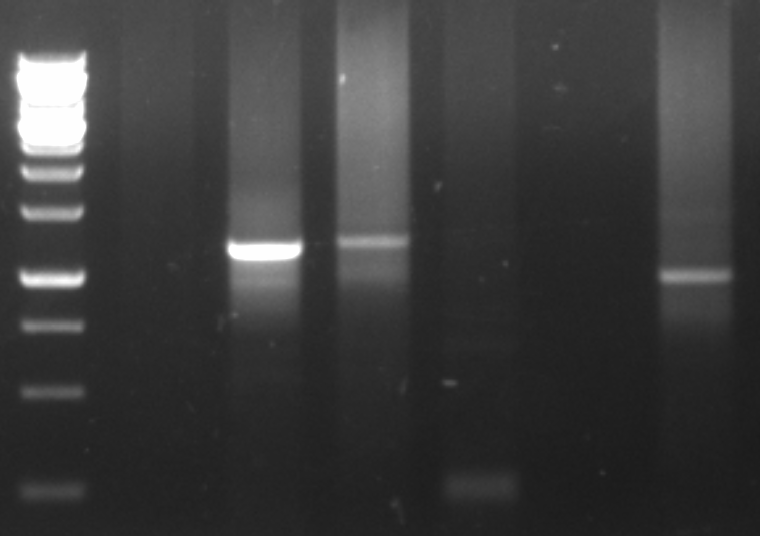

Supplement: Figure 7—figure supplement 1—source data 1. [file elife-103542-fig7-figsupp1-data1.zip › Figure 7-Figure Supplement 1-Source Data 1/raw agarose gels Fig7-supplement1 AB/Agarose gel Figure 7 - figure supplement 1B_PTP7-TGD in IT4var01-BirAP1_SLI2.tif]

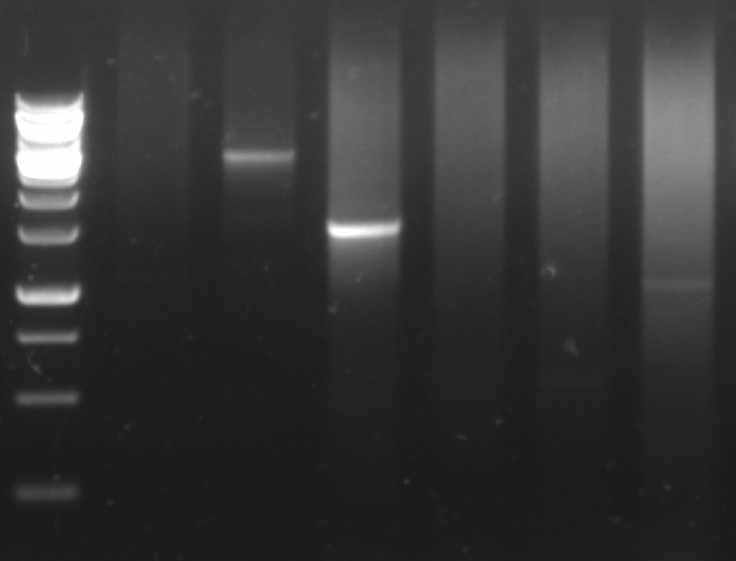

Supplement: Figure 7—figure supplement 1—source data 1. [file elife-103542-fig7-figsupp1-data1.zip › Figure 7-Figure Supplement 1-Source Data 1/raw agarose gels Fig7-supplement1 AB/Agarose gel Figure 7 - figure supplement 1B_TryThrA-TGD in IT4var01-BirAP1_SLI1.tif]

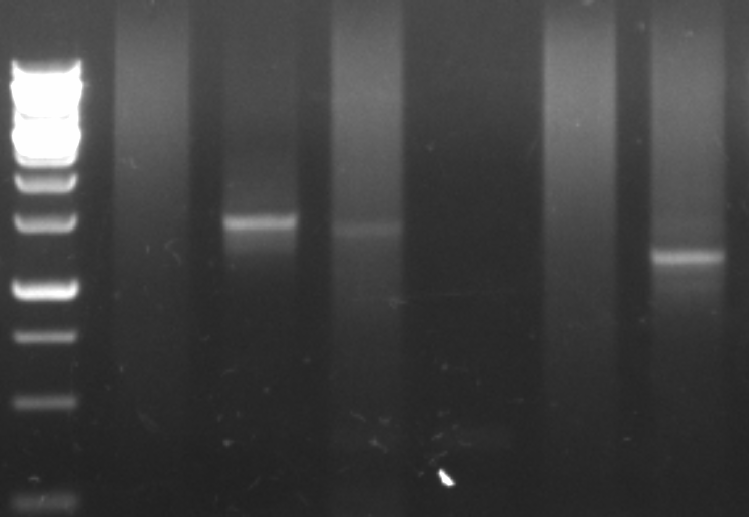

Supplement: Figure 7—figure supplement 1—source data 1. [file elife-103542-fig7-figsupp1-data1.zip › Figure 7-Figure Supplement 1-Source Data 1/raw agarose gels Fig7-supplement1 AB/Agarose gel Figure 7 - figure supplement 1B_TryThrA-TGD in IT4var01-BirAP1_SLI2.tif]

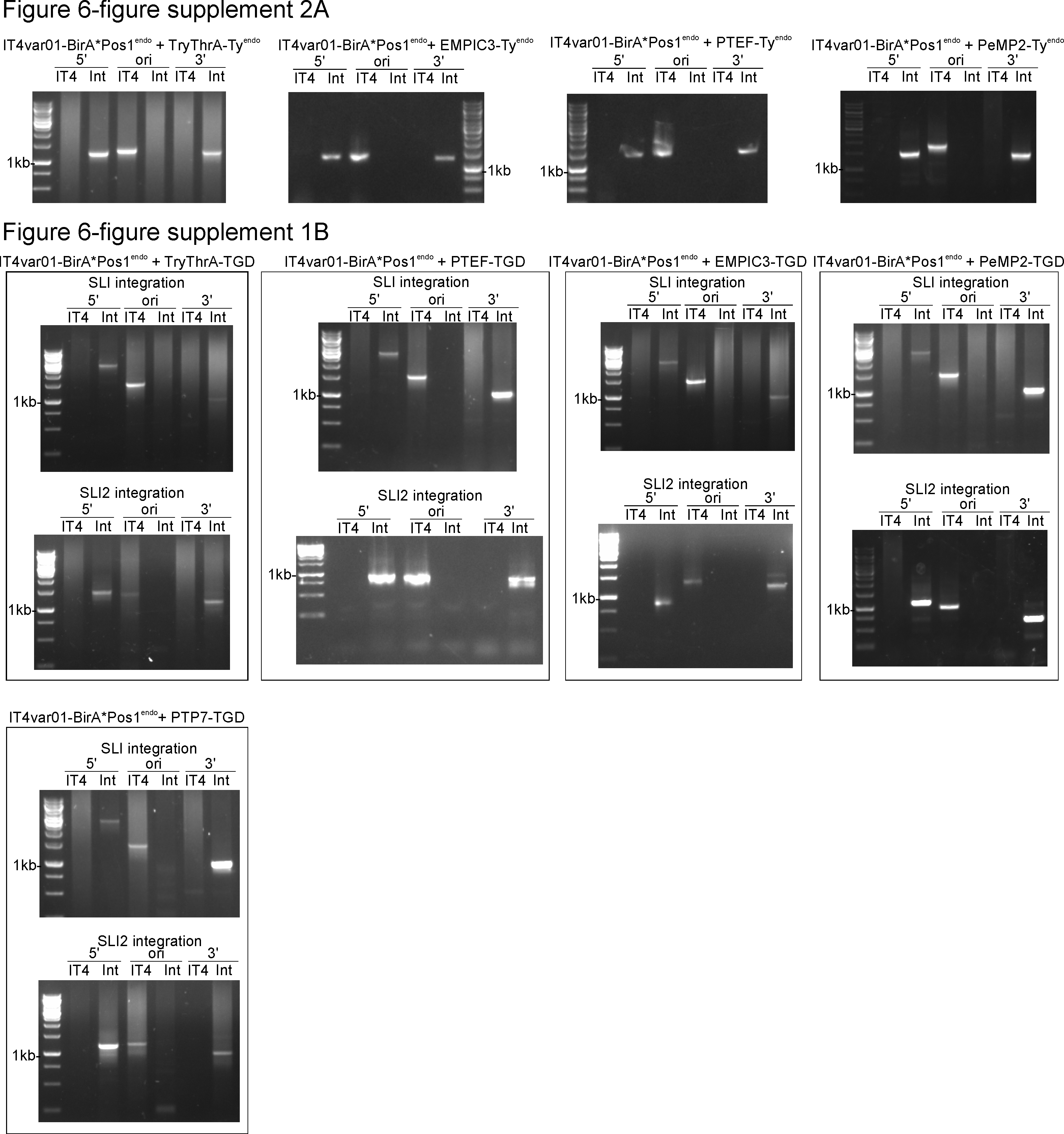

Supplement: Figure 7—figure supplement 1—source data 2. [file elife-103542-fig7-figsupp1-data2.zip › Figure 7-Figure Supplement 1-Source Data 2/annotated agarose gels Fig7-supplment1 AB/Figure 7-figure supplement 1_labelled.jpg]
